# HIV-1 BG505 SOSIP immunization induced B cell expansion targeting the 465-glycan hole, with neutralizing antibodies exhibiting distinct binding modes and mechanisms of virus inhibition

**DOI:** 10.1371/journal.ppat.1014268

**Published:** 2026-06-05

**Authors:** August Myers, Monika Chandravanshi, Leanne S. Whitmore, Brendan F. Kohrn, Amina Negash, Dung N. Nguyen, Pooja Ralli-Jain, Kendra Cruickshank, Amit A. Upadhyay, Tysheena Charles, Christopher T. Edwards, Eric Hunter, Rama R. Amara, Marek K. Korzeniowski, Ling Niu, Edwin Pozharski, William D. Tolbert, Steven E. Bosinger, Scott R. Kennedy, Marzena Pazgier, Cynthia A. Derdeyn

**Affiliations:** 1 Department of Laboratory Medicine and Pathology, University of Washington, Seattle, Washington, United States of America; 2 Infectious Disease Division, Department of Medicine, Uniformed Services University of the Health Sciences, Bethesda, Maryland, United States of America; 3 Henry M. Jackson Foundation for the Advancement of Military Medicine, Inc., Bethesda, Maryland, United States of America; 4 Washington National Biomedical Research Center, University of Washington, Seattle, Washington, United States of America; 5 Department of Pathology and Laboratory Medicine, Emory University, Atlanta, Georgia, United States of America; 6 Emory National Primate Research Center, Emory University, Atlanta, Georgia, United States of America; 7 Department of Microbiology and Immunology, Emory University, Atlanta, Georgia, United States of America; 8 Institute for Bioscience and Biotechnology Research, University of Maryland, Rockville, Maryland, United States of America; 9 Institute for Biochemistry and Molecular Biology, School of Medicine, University of Maryland, Baltimore, Maryland, United States of America; National Institute for Communicable Diseases, SOUTH AFRICA

## Abstract

High serum neutralization following BG505 SOSIP.664 envelope trimer immunization was associated with protection against BG505.SHIV challenge in rhesus macaques in a previous study. In an animal that developed high titer, durable neutralization against a glycan hole on envelope gp120, high throughput, longitudinal, antigen-specific B cell receptor sequencing was conducted. This analysis of more than 4,700 antigen-specific B cells revealed marked intra-clonal expansion and divergence from germline, including three abundant clonotypes that produced autologous neutralizing monoclonal antibodies. Monoclonal antibodies from the neutralizing clonotypes and two other expanded non-neutralizing clonotypes targeted epitopes in the same glycan hole, with neutralizers also demonstrating different capacities to obstruct CD4 binding. Cryo-electron microscopy structures of four neutralizing monoclonal antibodies revealed that they bound to glycan hole epitopes using distinct binding modes. One neutralizing antibody displaced a glycan in the loop V5 upon binding and its footprint includes the CD4 binding loop. The findings provide insight into how antibody recognition of a prominent glycan hole could facilitate different mechanisms of neutralization while underscoring how intra-clonal expansion and maturation with repeated BG505 SOSIP.664 immunization drove high serum neutralization.

## Author summary

BG505 SOSIP, a prototype stabilized soluble HIV-1 envelope trimer used broadly in vaccine studies, can elicit neutralizing antibodies associated with protection in nonhuman primate models. Here we demonstrate that profound intra-clonal expansion of antigen specific B cells accompanied the development of high titer, durable neutralizing antibody titers in an immunized rhesus macaque using high throughput longitudinal antibody variable domain sequencing of more than 4,700 antigen-specific B cells, immunologic assays, and structural studies. Of six highly abundant B cell clonotypes, five produced monoclonal antibodies that targeted the same site on BG505 envelope, and three produced monoclonal antibodies that neutralized BG505 virus. Cryo-EM structural studies revealed that the neutralizing monoclonal antibodies bound to distinct epitopes in the same region with unique binding modes that involved extensive interaction with the surrounding glycans and in one case, direct contact with the CD4 binding loop. Mechanisms of neutralization appear to include interference with envelope-CD4 binding. Together, these findings provide novel immunologic and mechanistic insights into modes of epitope recognition by SOSIP-elicited neutralizing antibodies and factors that favor the development of high and durable serum neutralization.

## Introduction

Robust protective vaccine efficacy against HIV-1 presents a challenge due to the genetic diversity and conformational complexity of the HIV-1 envelope (Env) glycoprotein, which is the only target on the virion surface to block entry into host cells [1]. Vaccine trials have not achieved significant protection against HIV-1 acquisition; however, antibodies that can neutralize globally circulating viruses have the potential to improve efficacy [2]. The development of stabilized, native-like HIV-1 Env gp140 trimers has provided significant advancements in vaccine design [1,3,4]. Trimer immunogens such as BG505 SOSIP.664 have elicited antibodies in rhesus macaques (RM) and humans that neutralized the matched virus *in vitro* and provided protection against autologous simian-human immunodeficiency virus (SHIV) challenge *in vivo* [5–9]. However, neutralizing antibodies (nAbs) elicited by stabilized trimers generally target ‘glycan holes’ present on Env gp120 [10–12]. These holes are unique to each Env, restricting nAbs to the matched virus and creating an impediment for neutralization breadth. Moreover, serum neutralizing ID_50_ titers vary widely even within the same study [5,7,9,10,13,14]. Finally, stabilized trimers also elicit antibodies that recognize neo-epitopes on the gp41 trimer base that are non-neutralizing [10,12,15].

In a previous study, RM were immunized with BG505 SOSIP.664 in 3M-052 adjuvant alone, or with three heterologous viral vectors (HVV) (vesicular stomatitis virus, vaccinia virus, adenovirus type 5) expressing only SIVmac239 Gag (S1 Table), which do not impact serum nAb titers [5,16]. Significant protection against repeated, low dose intra-vaginal SHIV.BG505 challenge was demonstrated with high serum neutralization ID_50_ titer emerging as one strong predictor of protection [5]. Using BG505 Env mutant pseudoviruses, we showed that serum nAbs in protected, high titer RM frequently targeted the glycan hole (465 GH) near the loop V5 on gp120 [10]. One animal in the HVV + SOSIP group, RUp16, developed serum nAb that targeted the 465 GH with an ID_50_ titer up to two logs higher than other animals in the cohort [5,10]. RUp16 serum neutralization did not appear to involve a different region on BG505 SOSIP.664 known as the 241/289 glycan hole [11], although serum antibodies against this site were detected in cryo-electron microscopy polyclonal epitope mapping (cryo-EMPEM) [10]. Together these observations suggested that the 241/289 targeted antibodies did not contribute appreciably to serum neutralization in RUp16, as cryo-EMPEM does not distinguish between neutralizing and non-neutralizing activity. A clonotype of monoclonal antibodies (mAbs) isolated from this animal neutralized BG505 Env pseudovirus by targeting the 465 GH [10]. This presented a well-characterized scenario with longitudinal sampling to examine how the dynamics of single antigen specific (Ag+) B cells during repeated immunization could lead to the robust development of nAbs.

Here we comprehensively characterized the high titer, durable nAb response in this RM using high throughput longitudinal Ag+ B cell receptor (BCR) sequencing combined with functional and high-resolution cryogenic electron microscopy (Cryo-EM) structural analyses of the corresponding mAbs. BG505 SOSIP.664 immunization drove an initially diverse Ag + B cell response; however, repeated immunization produced a subset of dominant clonotypes that underwent profound intra-clonal expansion and divergence from germline. The highly abundant neutralizing and non-neutralizing clonotypes we characterized frequently targeted the 465 GH, with hindrance of CD4 binding observed for some neutralizers. Structural studies revealed that the neutralizers used distinct binding modes, extensive glycan interactions, and indeed had epitopes that were proximal to or included elements of the CD4 binding site. The findings provide new insight into the immunogenicity and accessibility of this region to antibodies as well as varied potential mechanisms for 465 GH focused neutralization of BG505.

## Results

### Ag + B cells underwent marked intra-clonal expansion in response to repeated BG505 SOSIP immunizations

Previously, BG505 SOSIP.664 immunizations were administered in 3M-052 at weeks 16, 24, 40, and 80 interspersed with HVV-Gag at weeks 0, 8, and 36 [5]. The analysis presented here utilizes samples collected only after the first BG505 SOSIP.664 immunization at week 16 of the study (S1 Table). Ten weekly intravaginal challenges with BG505.SHIV ensued at week 84 and in a subset of protected animals, were followed by another 6 challenges beginning at week 114. One animal that remained uninfected after both challenge series, RUp16, developed serum ID_50_ neutralization titers that were the highest for this cohort [5,10]. The ID_50_ titer peaked at over 1:6,000 against the BG505 Env pseudovirus at week 84 and remained over 1:1,000 for another 37 weeks [5,10] and S1A and S1B Fig. In contrast, the median ID_50_ titer at week 84 for all other immunized animals was 1:61.

**Fig 1:**
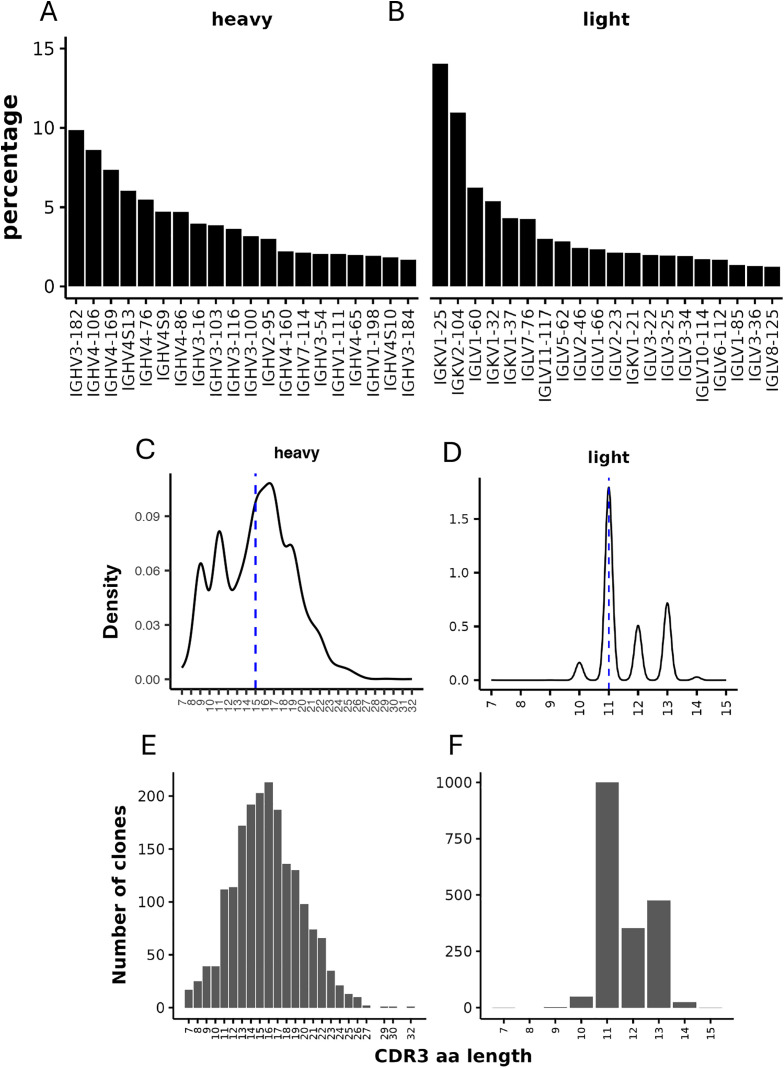
Overall characteristics of the Ag + B cell population. The top 20 heavy **(A)** and light **(B)** chain germline allele calls for each clonotype defined in this study are shown. The distribution of the amino acid lengths across all cells of the CDRH3 **(C)** and CDRL3 **(D)** are shown with the median length indicated (blue dashed line). The number of clonotypes with a given CDRH3 and CDRL3 amino acid length is shown in **(E)** and **(F)**.

10X Genomics BCR sequencing was performed using peripheral blood mononuclear cells (PBMC) samples collected from RUp16 at weeks 18, 20, 26, 36, 48, 49, 80, 81, 87, 93 and a lymph node (LN) fine needle aspirate (FNA) at week 83. A BG505 Env gp120 probe was used to sort Ag + B cells, as this approach had previously yielded a nAb producing B cell clonotype [10]. In total, 4,759 BCR sequences, consisting of paired heavy and light chains, were recovered with 1,901 clonotypes (including singlets) identified using scRepertoire v2.3.4 [17] and a custom RM immunoglobulin reference database augmented with individualized V_H_ gene germline alleles from this animal [18]. Fig 1A and 1B summarize the 20 most common V_H_ and V_L_ alleles in the Ag + B cell population, respectively. The length distribution of the third complementarity determining regions for V_H_ and V_L_, CDRH3 and CDRL3 across all cells, are shown in Fig 1C and 1D, respectively. The number of clonotypes that had CDRH3 or CDRL3 regions of a given length are shown in Fig 1E and 1F, respectively. Overall, the CDRH3 lengths from Ag+ B cells in this one immunized animal followed a similar distribution to antigen naïve and experienced human CDRH3 lengths [19]. The median CDRH3 length was 15 amino acids, ranging from 7 to 32 amino acids, while the CDRL3 lengths were more restricted (7–15 amino acids, median length of 11 amino acids).

**Fig 2:**
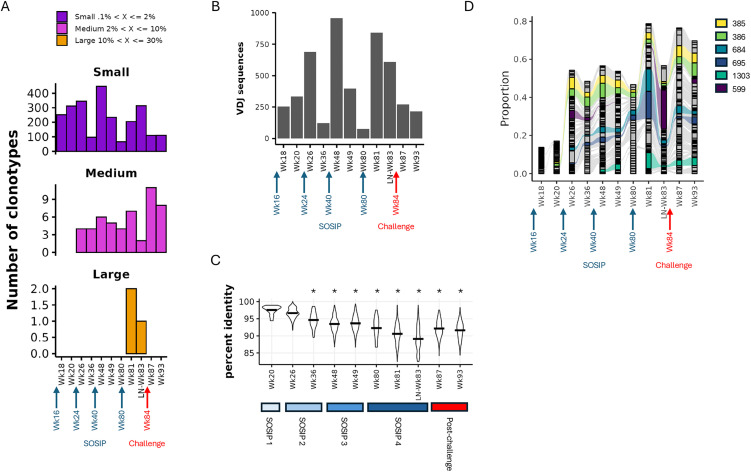
Ag + B cell dynamics with repeated BG505 SOSIP.664 immunizations. **(A)** Bar plot showing the number of clonotypes that appear in the population at each time point, categorized as small (purple, 0.1 to 2%), medium (pink, 2% to 10%) or large (orange, 10 to 30%). **(B)** Number of VDJ sequences recovered at each time point. **(C)** Recurring clonotypes that appeared in at least three of five periods were analyzed (n = 109; see S2 Fig). The percent identity of each sequence to the V_H_ germline call was determined and is indicated on the y-axis. Bars indicate the average percent identity shown within the distribution of values for each time point. An asterisk indicates a significant (adjusted P value < 0.05) difference for that time point compared to week 20 using the Wilcox rank sum test. **(D)** At each timepoint, the 30 most abundant clonotypes are stacked, with the size of each segment representing the proportion of the individual clonotype. The six large clonotypes of interest are color coded. The timeline of BG505 SOSIP.664 immunizations and SHIV.BG505 challenge is from [5]. ‘LN’ indicates a lymph node sample collected at week 83 while all others were PBMC samples.

Fig 2A groups clonotypes into three categories (small, medium, and large) based on their level of expansion at each time point. For example, a clonotype that was categorized as ‘medium’ would comprise between 2 and 10% of the population at that time point. This figure demonstrates that small clonotypes including singlets dominated the population at all time points, with medium and large clonotypes emerging after repeated SOSIP immunizations. The number of productive BCR sequences for each time point is shown in Fig 2B. This number tracked with expansion and contraction during repeated immunizations but was likely also affected by the cell number and viability of the sample. The responding B cells in RUp16 were initially diverse, consisting of only singlets and small clonotypes at week 18 and 20 (Fig 2A). After the second and third SOSIP immunizations, medium-sized clonotypes were observed at weeks 26, 36, 48, 49, and 80. Following the fourth immunization, large clonotypes emerged in circulation at week 81 and were in the LN at week 83. At 7 and 13 weeks after the last immunization (weeks 87 and 93), small and medium-sized clonotypes continued to persist but no large clonotypes were detected.

To investigate genetic divergence in the V_H_, we analyzed clonotypes that could be tracked longitudinally. Time points were categorized into 5 periods: post-SOSIP #1, #2, #3, #4, and the challenge phase. 109 clonotypes that were detected in at least 3 of the defined periods were included (S2A Fig). None of the clonotypes that met this criteria were observed at week 18. Fig 2C shows the percent nucleotide identity to the assigned V_H_ germline for each B cell within those clonotypes over time. Identity to germline was significantly lower than week 20 at all time points except week 26, with intra-lineage divergence from germline peaking in the LN sampled at week 83. Thus, B cell clonotype diversity at the antigen specific population level declined progressively as intra-clonal expansion occurred, during which time mutation away from germline increased intra-lineage diversity with repeated immunizations.

Fig 2D presents the dynamic behavior of the 30 most abundant clonotypes at each time point. Week 26 marked the onset of intra-clonal expansion while recall and persistence were observed and maintained throughout the immunizations and into the challenge phase. From this analysis, we focused on six abundant and recurring clonotypes within the top 30 that contained more than 100 individual B cells and were detected at most time points (S2B Fig). The neutralizing mAbs isolated in our previous study [10] belonged to clonotype 684 identified here. None of the large and durable clonotypes were detected at week 18 (Fig 2D); however, clonotypes 385 and 386 were detected as early as week 20 and at every subsequent time point. These six clonotypes were in the 30 most abundant lineages for the majority of time points, suggesting that they experienced significant advantages for survival and maturation *in vivo*. In the single LN analyzed at week 83, clonotype 599 was highly abundant; however, the other expanded clonotypes were also present in the top 30, demonstrating that they all trafficked between the periphery and this particular LN. Also, these six clonotypes were generally detected in the top 30 even when the total number of sequences was low (weeks 36, 80). Overall, profound intra-clonal expansion of a subset of Ag + B cell clonotypes occurred in parallel with the development of high and durable serum nAbs.

### mAbs from three large B cell clonotypes neutralize BG505 Env pseudovirus

**Fig 3:**
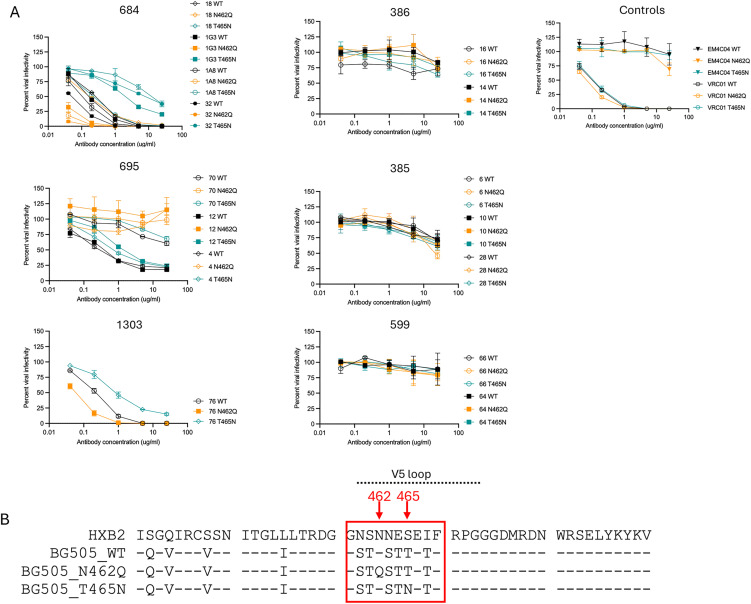
Neutralization BG505 Env pseudoviruses in the TZM-bl assay. In each graph, the percent virus infectivity is shown on the y axis plotted against the mAb concentration on the x-axis on a log10 scale. Serially diluted mAb at 25, 5, 1, 0.2, and 0.04 μg/mL was tested. **(A)** Each antibody was tested against BG505 WT (black), N462Q (orange), and T465N (teal) Env pseudoviruses. The corresponding IC_50_ values are shown in Table 1. Each mAb-Env combination is shown in the legend for each graph. The clonotypes are indicated above each graph. Positive (VRC01) and negative (EM4C04, anti-flu HA) antibodies are shown in a separate graph. **(B)** An alignment shows the region of Env where the N462Q and T465N changes are located, with BG505 WT and HIV-1 HXB2 Envs provided for reference. Residues that are conserved with HXB2 are dashes while differences are shown. The loop V5 is indicated.

We first generated representative mAbs from the six clonotypes and evaluated their binding to BG505 Env gp120 or BG505 SOSIP.664 using biolayer interferometry (BLI). All mAbs tested bound to gp120 with kinetics similar to the CD4 binding site (CD4bs) broadly neutralizing antibody (bnAb) VRC01 (S3A and S3B Fig, left columns). mAbs from five of the six clonotypes also bound comparably to BG505 SOSIP.664; however, clonotype 599 mAbs bound poorly to the SOSIP (S3A and S3B Fig, right columns). Next, each mAb was tested for neutralization of BG505 Env pseudovirus in the TZM-bl assay. mAbs from clonotypes 684, 695, and 1303 neutralized BG505 Env pseudovirus with IC_50_ titers in the range of 0.05 to 0.42 μg/mL (Table 1 and Fig 3A). For clonotype 684, we included two mAbs isolated previously from PBMC through single Ag + B cell sorting into 96 well plates followed by RT-PCR (1G3 from week 44 [10] and 1A8 from week 84) alongside two antibodies generated here from the 10X Genomics VDJ sequences at weeks 26 and 93. Clonotype 684 was first detected at week 26, and the mAb from that time point (mAb 18) neutralized BG505 Env pseudovirus with an IC_50_ titer of 0.24 μg/mL despite having 98% nucleotide identity to germline (Table 1 and Fig 3A). Neutralization potency increased 5-fold between weeks 26 and 93 to 0.05 μg/mL, along with substantial divergence from both V_H_ and V_L_ germline alleles. For clonotype 695, mAb 70 isolated from week 26 was weakly neutralizing (Table 1 and Fig 3A). The clonotype 695 mAbs from weeks 48 and 81 neutralized better with higher divergence from germline, reaching IC_50_ titers of 0.39 and 0.42 μg/mL, respectively. Additionally, the neutralization curve of the clonotype 695 antibodies 4 and 12 against wild type (WT) BG505 and T465N exhibited a plateau that can be caused by factors such as virion heterogeneity, low stoichiometry, and high off antibody rates [2,20,21] (Fig 3A). For the third neutralizing clonotype, 1303, only one mAb was tested, from week 81. This mAb 76 had an IC_50_ titer of 0.21 μg/mL, similar to the potency of mAbs from clonotypes 684 and 695. The V_H_ and V_L_ germline alleles and CDRH3 lengths were not shared across the neutralizing clonotypes (S2B Fig). In contrast, despite robust intra-clonal expansion and persistence, none of the mAbs tested from clonotypes 385, 386, or 599 neutralized the BG505 Env pseudovirus (Table 1 and Fig 3A). We note that mAbs from clonotype 385 and 386, while non-neutralizing, showed robust binding to BG505 SOSIP.664 (S3A Fig, right column). The 599 mAbs, however, bound better to gp120 despite their abundance in the week 83 LN, suggesting that the trimer may not have remained completely intact *in vivo*. Taken together, the results indicate that antibody binding to the soluble trimer does not necessarily predict neutralization of the virus.

**Table 1:** Neutralization data and identity to germline for selected RUp16 mAbs.

			IC_50_ μg/mL			Mutant/WT		percent identity to GL	
**Clone**	**mAb**	**Time point (wks)**	**WT**	**N462Q**	**T465N**	**N462Q**	**T465N**	**VH**	**VL**
**684**	**18**	26	0.24	0.18	12	0.7	51	98	98
	**1G3**	44	0.18	0.02	2.2	0.1	12	94	95
	**1A8**	84	0.11	0.01	0.23	0.1	2.1	90	92
	**32**	93	0.05	0.01	7.9	0.2	163	85	91
**695**	**70**	26	25	25	25	1.0	1.0	96	98
	**12**	48	0.39	25	2.0	65	5.1	90	97
	**4**	81	0.42	25	1.1	60	2.6	88	88
**1303**	**76**	81	0.21	0.06	1.0	0.3	4.8	83	94
**386**	**16**	26	25	25	25	1.0	1.0	87	92
	**14**	48	25	25	25	1.0	1.0	88	88
**385**	**6**	20	25	25	25	1.0	1.0	97	92
	**10**	87	25	25	25	1.0	1.0	95	90
	**28**	93	25	25	25	1.0	1.0	94	89
**599**	**66**	26	25	25	25	1.0	1.0	94	97
	**64**	93	25	25	25	1.0	1.0	87	93
**Pos**	**VRC01**	NA	0.10	0.06	0.11	0.6	1.1	68	83
**Neg**	**EM4C04**	NA	25	25	25	1.0	1.0	NA	NA

Neutralization IC_50_ titers of mAbs evaluated in the TZM-bl assay are shown against the wildtype BG505 Env pseudovirus and BG505 Env pseudoviruses containing the N462Q and the T465N substitutions. The fold change (mutant divided by wildtype IC_50_) is shown. The clonotype, time point of isolation, and identity to the V_H_ and V_L_ germline alleles are also indicated for each mAb. For IC_50_ titers, shading indicates potency, with green colors indicating higher potency and red indicating resistance at the highest concentration tested (25 μg/mL). For mutant vs. WT comparison, values greater than 1 indicate that the mutant was less susceptible to mAb neutralization than the WT, with larger differences indicated by shaded bars. Values less than 1 indicate that the mutant was more sensitive than the WT to the indicated mAb.

### The glycan at N462 near the CD4bs impacts neutralization of BG505 Env pseudovirus

Previously, mAb 1G3 neutralization was mapped to the 465 GH using multiple mutant BG505 Env pseudoviruses including T465N [10]. Removal of the glycan motif at N462 in loop V5 was not included in that previous study but it is proximal to the 465 GH and modulates access to the CD4bs [22–24]. We therefore generated an N462Q mutant BG505 Env pseudovirus (Fig 3B) and evaluated neutralization compared to wildtype in the TZM-bl assay. The N462Q mutant BG505 Env pseudovirus was more sensitive than the wild type to mAbs from clonotypes 684 and 1303 (Table 1 and Fig 3A). In contrast, the N462Q pseudovirus was more resistant than wild type to neutralization by the later antibodies from clonotype 695. These results suggest that for clonotypes 684 and 1303, a glycan at N462 partially obstructed epitope access. Clonotype 695 mAbs were different in that they appear to require the N462 glycan for neutralization. mAbs that could not neutralize the wild type BG505 Env pseudovirus were also not able to neutralize N462Q, suggesting that this glycan did not impede access to their epitopes (Table 1 and Fig 3A). For additional insight into the neutralization targets, we also tested the mAbs against a BG505 Env pseudovirus with the T465N substitution, which introduces a glycan near the 465 glycan hole (Fig 3B) [10,11]. mAbs from the 684 clonotype showed variable sensitivity to T465N (Table 1 and Fig 3A), consistent with our previous results that included mAb 1G3 [10]. The increase in IC_50_ titer ranged from 2.1-fold (mAb 1A8) to 163-fold (mAb 32). The other neutralizing mAbs (mAbs 4, 12, and 76) had modest increases in IC_50_ against T465N ranging from 2.6- to 5.1-fold. None of the non-neutralizing mAbs were affected by the T465N mutation. This data supports that the neutralizing mAbs bind epitopes proximal to the 465 glycan hole but they are differentially impacted by addition and removal of these loop V5 proximal glycans [10].

### Neutralizing mAbs from two clonotypes have the potential to hinder Env-CD4 interactions at varying levels

**Fig 4:**
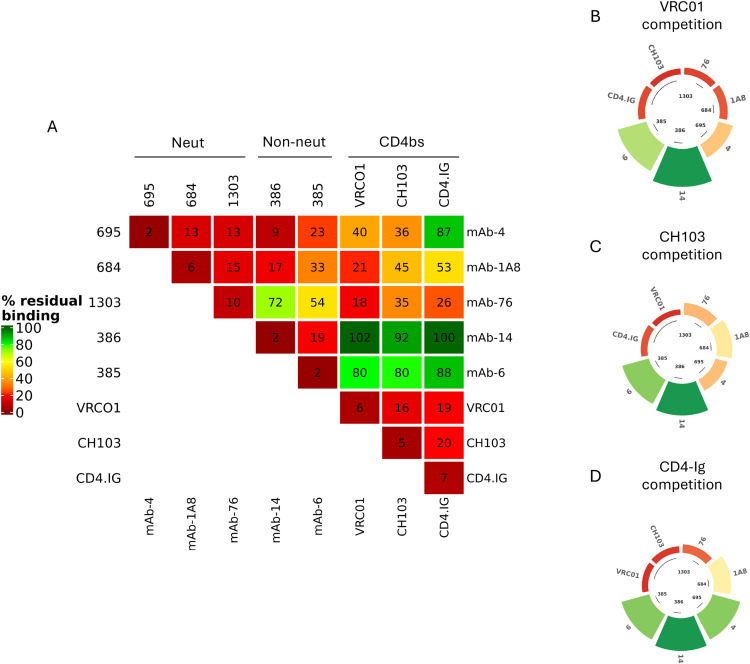
Competition matrix for binding to BG505 gp120 using BLI epitope binning. **(A)** Competition is represented as the average percent residual binding of the secondary antibody or CD4-Ig in the presence of the primary antibody or CD4-Ig compared to no primary reagent. The mAbs were tested in both directions with the results averaged. Recombinant human CD4-Ig was used to test for binding to elements of the CD4bs; bnAbs VRC01 and CH103 were used to test for binding proximal to the CD4bs; EM4C04 (anti-flu HA) was used as a negative control that does not bind HIV Env. mAb IDs are indicated along the right and the bottom of the matrix; clonotypes are indicated on the left and top. Residual binding is shown on a color gradient from 0% (red) to 100% (green) with the value indicated in each box. Reduction of VRC01 **(B)**, CH103 **(C)**, and CD4-Ig **(D)** binding by each antibody or CD4-Ig is depicted by the height and color of each segment, corresponding to **(A)**. Clonotype numbers are shown on the inside; mAbs and CD4-Ig are indicated on the outside. mAbs 76, 1A8, and 4 (clonotypes 1303, 684, 695) are neutralizers. mAbs from clonotype 599 were excluded from this analysis as they did not bind to gp120-His when immobilized on the sensor.

Competition (binning) experiments were performed using BLI to determine whether mAbs from different clonotypes recognized proximal or distal epitopes from one another as well as the CD4bs. The C-terminally His-tagged BG505 Env gp120 protein was immobilized to a Ni-NTA sensor and tandem competitions were performed using pairs of primary and secondary antibodies. mAbs from clonotype 599 bound poorly to BG505 Env gp120 in this configuration and were excluded from the competition analysis. To investigate whether (i) the expanded clonotypes were targeting a proximal region on Env and (ii) a potential mechanism of neutralization could be interfering with Env-CD4 interactions, we selected one mAb from each of the five clonotypes for a competition matrix that included the CD4bs bnAbs VRC01 [25] and CH103 [26] and recombinant human CD4-Ig protein. A larger competition matrix is shown in S4A Fig. VRC01 and CH103 had similar competition profiles against the vaccine-elicited mAbs and CD4-Ig (Fig 4A). The neutralizing mAbs 4, 1A8, and 76 competed against each other, and mAbs 4 and 1A8 also competed with the non-neutralizing mAbs 14 and 6. The neutralizing mAbs also competed to varying degrees against the CD4bs bnAbs but only mAb 76 competed against CD4-Ig. The two non-neutralizing mAbs, 6 and 14, competed weakly or not at all with the CD4bs bnAbs and CD4-Ig. These data suggest that the neutralizing mAbs recognize proximal epitopes that are in some cases near the non-neutralizers, and that the neutralizers have distinct abilities to compete against the CD4bs bnAbs and CD4-Ig (Fig 4B, 4C, and 4D). All of the neutralizing mAbs we tested competed against VRC01 and/or CH103, indicating some proximity to the CD4bs (Figs 4A, S4A and S4B). Taken together, these findings raise the possibility that some of the neutralizing epitopes are proximal to the CD4bs while the epitope for mAb 76, which competes with CD4-Ig, includes elements of the CD4bs. Furthermore, they suggest that mAb 76 could neutralize BG505 pseudovirus by directly interfering with Env-CD4 engagement, while mAbs such as 1A8 could neutralize by sterically inhibiting this process [2,15]. On the other hand, mAb 4 likely neutralizes BG505 pseudovirus through a CD4-independent mechanism, such as slowing entry of adsorbed virions [27] or inter-trimer crosslinking on the virion [2,28], which have been attributed to V3-high mannose binding site bnAbs. Within a single immunized animal, three clonotypes that targeted the 465 GH likely utilize distinct mechanisms of virus neutralization, while two clonotypes in or near this region fail to neutralize, highlighting the complexity and heterogeneity within this immunogenic target.

### Structural definition of neutralization epitopes within a CD4bs proximal glycan hole

To refine our understanding the neutralization mechanisms of BG505 SOSIP.664–elicited antibodies, along with epitope overlap and proximity to the CD4bs, we selected four mAbs and solved cryo-EM structures of their Fabs in complex with BG505 SOSIP.664 and PGT121 Fab as a chaperone. These include mAbs 1G3 and 1A8 from clonotype 684, mAb 4 from clonotype 695, and mAb 76 from clonotype 1303. Cryo-EM structures were solved at resolution range of 2.98 to 3.64 Å (S4 Table and S5 Fig).

**Fig 5:**
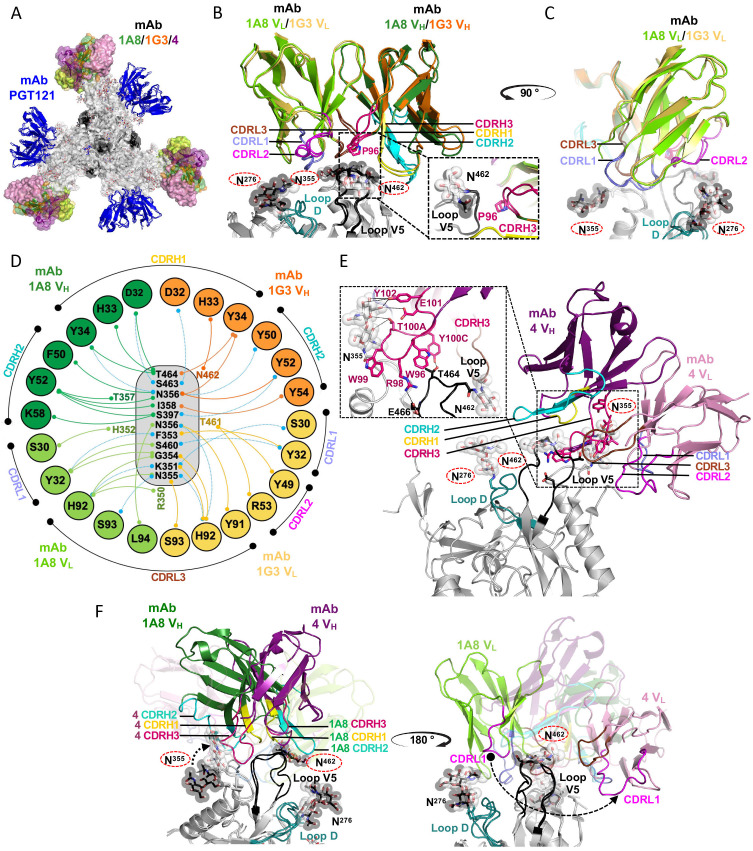
Structural basis of 465GH-guided epitope recognition by mAbs 1G3, 1A8, and 4. **(A)** Top view of Env trimer (gp120 in grey, gp41 in black) complex with Fabs 1A8 (green), 1G3 (orange), 4 (purple) and chaperone Fab PGT121 (blue). The variable domains are shown as dark and light shaded surfaces for V_H_ and V_L_ chain, respectively. **(B)** Zoomed view showing contacts between variable domains of mAbs 1A8 and 1G3 with the gp120 protomer. A central proline introduces a kink in CDRH3 that is highlighted in the blow-up view. Epitope glycans are shown as sticks and spheres. **(C)** Side-view orientation of mAbs 1A8 and 1G3, highlighting the close proximity of their light chain CDRs to gp120 loop D (cyan) region. **(D)** Interaction network between mAb 1A8 or 1G3 (outer circle) residues and the adjacent gp120 protomer residues (inner grey circle), defined by a 4 Å cutoff. Dashed blue lines represents hydrogen bonds. **(E)** Zoomed view depicting mAb 4’s angle of approach with the gp120 protomer. Red dotted circles highlight epitope glycans. Contacts between the aromatic-rich region of CDRH3 and the N355 glycan are highlighted in a blow-up view. **(F)** Structural superimposition reveals a distinct angular approach of Ab4 V_H_ (purple) relative to 1A8 V_H_ (green) on gp120 (grey) and shows ~180° rotation of Ab4 V_L_ relative to 1A8 V_L_.

**Fig 6:**
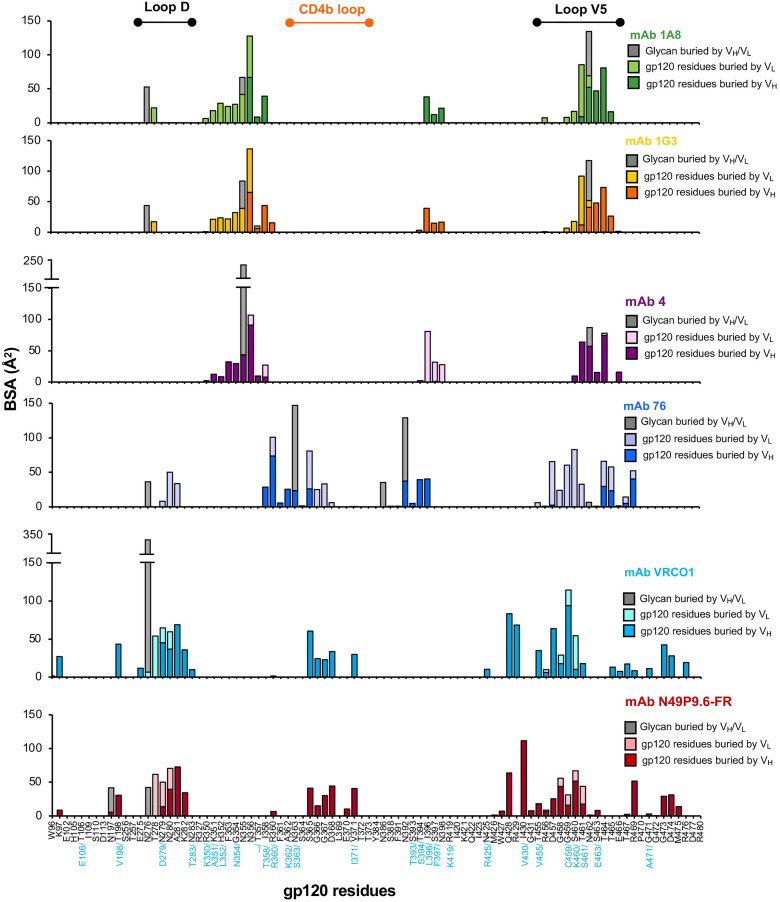
Buried surface area (BSA) of mAbs within the SOSIP.664 complex interface. BSA of the Env antigen as a function of residue contributed by mAbs 1A8, 1G3, 4, 76, VRC01 (PDB id: 5FYJ) and N49P9.6-FR (PDB id: 7UOJ) [23,29] as calculated by PISA [30], shown as bars. The y-axis represents the buried surface area (BSA), while the x-axis lists gp120 residues from BG505 SOSIP.664. Residues in gp120 of X1193.c1 SOSIP.664 that differ from the BG505 SOSIP.664 sequence are highlighted in blue along the x-axis. The BSA is divided into separate contributions by V_H_ and V_L_ for protein (shown in darker and lighter shades, respectively), but combined contributions by Fab for glycan (shown in grey).

Shown in Fig 5A are the overall structures and detailed views of the epitopes and antigen–Fab interfaces for mAbs 1A8 and 1G3 (clonotype 684) and mAb 4 (clonotype 695). These antibodies recognize overlapping epitopes within the 465 GH of the gp120 outer domain. As expected, mAbs 1G3 and 1A8 bind their epitopes in a highly similar manner, engaging almost the same gp120 regions formed by loops D, V5, and the loop connecting the α2 helix–β14-strand with a comparable number of contacts between the antibody antigen-binding site and the gp120 epitope (Fig 5B-5D). The 465 GH is surrounded by glycans on N276, N339, N355, N386, N392, and N462 but only three of them, N355 (loop connecting the α2 helix to the β14-strand), N462 (loop V5), and N276 (loop D), contribute to 1G3 and 1A8 binding as determined by buried surface area (BSA) calculations (Figs 5B and 6). Both mAbs have a short nine-amino-acid CDRH3 that forms minimal contact with gp120, while the other CDRs (CDRL1–3 and CDRH1–2) extend outward to engage both protein residues and glycan moieties. In the short CDRH3, a central proline residue is completely conserved within clonotype 684, and likely forms a kink at its tip to reorient and accommodate gp120 loop V5 as is seen in both complex structures (Fig 5B, zoomed region). CDRH1 contacts the glycan on N462 while CDRL2 and CDRL3 interact with glycans on N276 and N355, respectively (Fig 5B and 5C). Collectively, this suggests that recognition is primarily mediated through protein contacts after glycan rearrangements to allow access to the underlying epitope core.

To test whether 1G3 and 1A8 rely more on protein and not on glycan moieties for binding, we performed SPR using BG505 SOSIP.664 immobilized on a sensor chip. Two variants were tested: wild-type BG505 SOSIP.664 carrying complex glycans and a glycan-trimmed variant with sugars reduced to the GlcNAc core after Endo H treatment (Table 2 and S6 Fig). As anticipated, both Fabs bound more efficiently to the glycan-trimmed Env. The binding affinity (K_D_) for 1G3 and 1A8 with wild type BG505 SOSIP.664 were 16 and 12 nM, respectively. However, when the Endo H treated SOSIP was used, we observed 3.2 and 4.2-fold faster association rates and increases in the binding affinity by 14 and 15-fold for 1G3 and 1A8, respectively (K_D_s of 1 and 0.8 nM, respectively) (Table 2 and S6 Fig). mAb 1A8, isolated 40 weeks later than 1G3, is more somatically mutated and further from germline sequences than 1G3 (Table 1) which seems to have increased its affinity but not its complex interface BSA or neutralization potency. These changes could have stabilized 1A8 binding, as its neutralization potency is less affected by the T465N substitution than 1G3 (Table 1 and Fig 3A). The total BSA of the 1A8 Fab to the interface is 892 Å^2^ (392 and 357 Å^2^ from the V_H_ and V_L_ to the Env protein, respectively, and 143 Å^2^ from both chains to the three epitope glycans) as compared to 909 Å^2^ for the 1G3 (406 Å^2^ and 350 Å^2^ from the V_H_ and V_L_ to the Env protein and 153 Å^2^ to the three epitope glycans) (Fig 6).

**Table 2:** Kinetic parameters of Fab binding with BG505.SOSIP.664 calculated from SPR analyses.

Fab	BG505.SOSIP	k_on_ (x10^3^)	k_off_ (x 10^–6^)	K_D_ (nM)
1A8	Expi	7.0 ± 0.4	82 ± 18	12 ± 2
	GnTI^-^-EndoH	30 ± 4	23 ± 6	0.8± 0.2
	**Fold change**	**4.2**	**3.5**	**15**
1G3	Expi	6.4 ± 0.8	101 ± 7	16 ± 0.9
	GnTI^-^-EndoH	20 ± 17	29± 7	1 ± 0.4
	**Fold change**	**3.2**	**3.5**	**14**
4	Expi	14± 0.5	89± 14	6.5± 0.9
	GnTI^-^-EndoH	69± 9	66± 14	0.9± 0.09
	**Fold change**	**5.0**	**1.4**	**7**
76	Expi	3± 0.8	149± 51	50± 4
	GnTI^-^-EndoH	9± 0.1	34± 5	4± 0.6
	**Fold change**	**3**	**4.4**	**13**
8ANC195	Expi	3± 0.5	336± 40	119± 10
	GnTI^-^-EndoH	3± 0.9	247± 41	98 ± 15
	**Fold change**	**0.9**	**1.4**	**1.2**
N49P9.6-FR	Expi	52± 1	67± 2	1.3± 0.07
	GnTI^-^-EndoH	510 ± 68	15± 3	0.03 ± 0.002
	**Fold change**	**10**	**5**	**44**

The on rate (K_on_), off rate (K_off_) and equilibrium dissociation constant (K_D_) are shown for each mAb tested using glycosylated or de-glycosylated BG505 SOSIP.664, including the fold change.

mAb 4 was isolated from clonotype 695 at week 81, similar in time point to 1A8 at week 84 (Table 1). The structure of the complex of Fab 4 with BG505 SOSIP.664 revealed that it recognizes an epitope within the same 465 GH region as recognized by 1G3 and 1A8 (Fig 5E), but with a distinct binding mode and slightly altered epitope footprint. The most noticeable differences are in the angle of approach of mAb 4 to the trimer as compared to 1A8 and 1G3 and in the conformations of loops D and V5 between the complexes, which are evident when Fab 1A8 and Fab 4 are superimposed based upon gp120. Most importantly, Fab 4 engages the 465 GH region by swapping the relative positions of its light and heavy chains as compared to 1A8 (Fig 5F). Via this swap, the Fab 4 CDRH3 makes extensive contacts with gp120 and the glycan on N355, while CDRH1 and CDRH2 contact the glycan on N462 (S8A Fig). In addition, the Fab 4 CDRH3 is enriched in aromatic residues, enabling it to establish extensive contacts with the glycan at N355 (Figs 5E zoomed region, and S8A). By reorienting the N355 glycan, the CDRH3 facilitates access to the underlying protein epitope (Fig 5F) without contacting loop D or the glycan on N276. Extensive contact to the N355 glycan is characteristic for mAb 4 and likely adds to its ability to neutralize BG505 Env pseudovirus (Table 1 and Fig 3A). Fab 4’s V_L_ only makes minimal contact with Env antigen. Overall, Fab 4 buries 875.6 Å² of protein surface area at the interface, with 474.7 Å² and 180.8 Å² contributed by the V_H_ and V_L_, respectively. Both chains together bury an additional 220 Å² on glycans N355 and N462. This suggests that unlike 1G3 and 1A8, mAb 4 depends less on glycan rearrangement to access the GH epitope. This is further supported by smaller changes in the binding kinetics of mAb 4 to wild type and glycan-trimmed SOSIPs (Table 2 and S6 Fig). Although glycan removal enhances the association constant of mAb 4 by about 5-fold, the overall binding affinity showed only a modest change, with about 7-fold change in K_D_ (K_D_ values of 6.5 nM and 0.9 nM for wildtype and glycan-trimmed, respectively). However, in the context of the pseudovirus, mAb 4 neutralization appears to be dependent on N462, possibly to stabilize its binding (Table 1 and Fig 3A). For comparison, we also measured the binding affinity of bnAb 8ANC195, which binds at the gp120-gp41 interface and only marginally depends on relocation of N-linked glycans [31] (Table 2 and S6 Fig). As expected, like mAb 4, 8ANC195 only shows small changes in the association constant (0.9 fold) and K_D_ values (1.2-fold) when interacting with wild type and glycan-trimmed BG505 SOSIP.664. Finally, the epitope footprints of mAbs 1G3, 1A8, and 4 include Env outer domain residues 350–358, loop V4 residues 396–398, and loop V5 residues 459–466 which are not conserved across variants. Consistent with this, two antibodies from the 684 clonotype that includes mAbs 1G3 and 1A8 lacked heterologous neutralization [10], as does mAb 4 (S7 Fig). Thus, all three antibodies rely on strain specific contacts within the exposed GH that limit neutralization activity to BG505.

**Fig 7:**
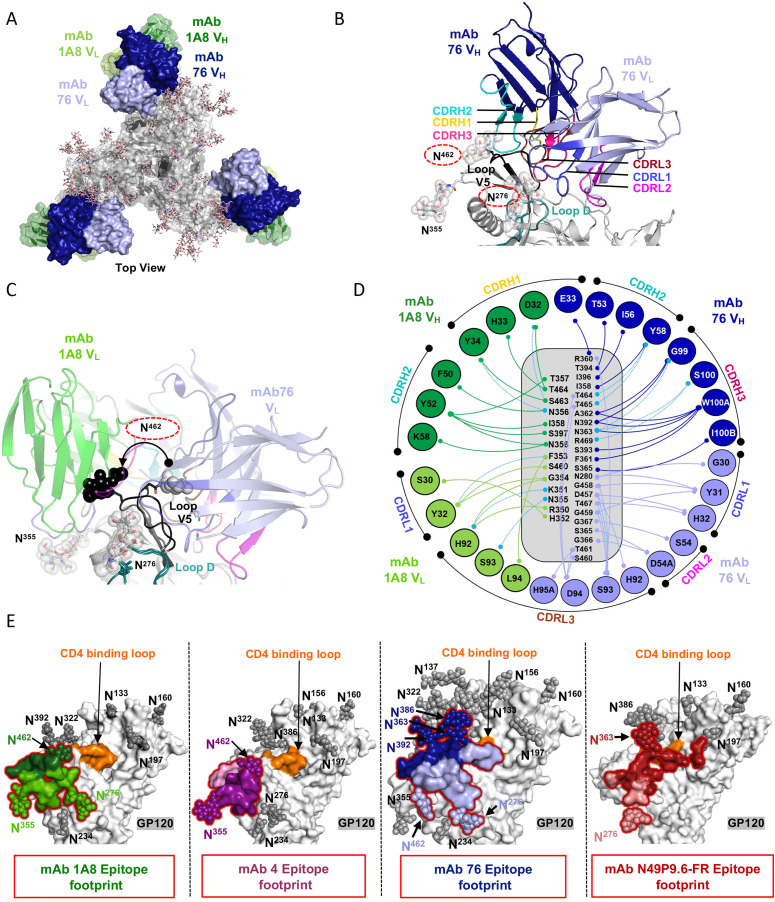
Structure of Fab 76 in complex with BG505 SOSIP.664. **(A)** Structural comparison of the Fab 76-BG505 SOSIP.664 complex illustrates its distinct binding mode as compared to the Fab 1A8-BG505 SOSIP.664 complex. V_H_ and V_L_ are colored darker and lighter surfaces, respectively. **(B)** Zoomed view of the Fab 76 and gp120 interface. Epitope glycans are displayed in sticks and spheres. **(C)** Structural overlay of Fab 76 and 1A8 complexes showing their lack of overlap and the conformational change of loop V5 (black) which is accompanied by the change in position of the N462 glycan. **(D)** Comparative interaction networks of 1A8 and 76 Fab residues (outer circle) with gp120 (inner grey circle), mapped in a 4 Å cutoff criterion. Dashed blue lines represent hydrogen bonds. **(E)** Epitope footprints of Fabs are shown as colored surfaces on the BG505 SOSIP.664 with the CD4 binding loop highlighted in orange. Epitope-associated glycans are represented as colored spheres and other glycans as grey spheres. Epitope footprint edges are outlined in red and the contribution of the footprint by the heavy and light chains shown in darker and lighter shades, respectively.

The most interesting antibody was mAb 76, from clonotype 1303 (isolated at week 81). Like mAbs 1G3, 1A8, and 4, mAb 76 also binds within the 465 GH in the outer domain region of gp120 (Fig 7A); however, its fine epitope footprint is significantly different (Fig 6). While preserving contacts to loop D and V5, mAb 76 shifts its epitope more toward the CD4 binding cavity and CD4 binding loop (Fig 7B-7E), which is consistent with its ability to obstruct CD4-Ig binding to BG505 Env gp120 (Fig 4). Fab 76’s V_L_ makes extensive contact with loop V5 of gp120, predominantly through CDRL1 and CDRL3 (Fig 7D). In particular, Tyr31 from CDRL1 bridges loops D and V5 by interacting with Env Asn280 and Gly459 (S9A Fig). This repositions the N462 glycan relative to its orientation in the 1A8 and 1G3 complexes such that N462 now directly contacts Fab 76’s CDRH3 (Fig 7B and 7C). Overall, compared to other clonotypes, mAb 76 engages a broader set of the surrounding glycans, including those on N276, N363, N386, N392, and N462 (Fig 7E). mAb 76 buries 1306.2 Å² on the gp120 protein core, with contributions of 411.2 Å² and 601.4 Å² from the V_H_ and V_L_, respectively, and an additional 293.7 Å² from glycans (attached to N276, N363, N386, N392, and N462). However, similar to mAbs 1A8, 1G3, and 4, glycans probably initially restrict accessibility and glycan contacts are likely established after penetration of the glycan shield. Consistently, SPR analyses show that mAb 76 binds more efficiently to the deglycosylated BG505 SOSIP.664 with an ~ 3-fold increased association rate and an ~ 13-fold higher affinity (K_D_ of 4 nM as compared to a K_D_ of 50 nM for the glycosylated trimer) (Table 2 and S6 Fig). Importantly, mAb 76 navigates through the surrounding glycans to engage loop D, the CD4 binding loop, and conserved CD4 binding contacts proximal to loop V5 (Figs 6 and 7E). Its epitope also partially overlaps with CD4bs bnAbs such as VRC01 and N49P9.6-FR [29] (Figs 6, 7E and S9B). We extended this analysis to a broader range of CD4bs antibodies, specifically 04_A06, VRC13, 3BNC117, and CH103 [32,33], to assess epitope similarity (S10 Fig) as well as the conservation of residues within epitopes (S11 Fig). mAb 76’s overlap with CD4bs antibodies resides mostly within the CD4 binding loop and to the N276 glycan, but its V_L_ also blocks access to the CD4bs cavity (Fig 7E). While mAb 76 does utilize conserved residues within the CD4bs it still relies on surrounding residues that are less conserved as do mAbs 1G3, 1A8 and 4 (S11 Fig). bnAb N49P9.6-FR bound more tightly to both glycosylated and deglycosylated SOSIP.664 trimer than the other mAbs from this study suggesting its higher affinity to SOSIP.664 trimer is also a component of its neutralization breadth (Table 2 and S6 Fig). To assess the functional relevance of the overlap with the CD4bs, we evaluated the neutralizing activity of mAb 76 against a global panel of HIV-1 Env pseudoviruses. Unfortunately, it did not neutralize heterologous viruses (S7 Fig). Thus, while mAb 76 engages conserved elements of the CD4bs, its dependence on BG505-specific residues that are not conserved limits its breadth and accounts for its strain-restricted neutralization profile.

## Discussion

Here we used high resolution, longitudinal Ag + B cell dynamics, functional antibody analysis, and antibody-antigen structural determinations to understand why a RM immunized four times with BG505 SOSIP.664 developed high and durable autologous serum nAb that remained elevated for 40 weeks post-immunization [5,10]. The in-depth characterization of such favorable responses to BG505 SOSIP.664 immunization in a highly relevant model is important and informative as trimer constructs form the basis for next generation vaccines and clinical advancement [9,13,34,35]. Indeed, a recent study in which RM were vaccinated sequentially with different trimers described elicitation of mAbs with heterologous neutralization [36]. Our study also highlights possible obstacles to acquiring neutralization breadth despite expansion, trafficking, and antibody mutation. In this BG505 SOSIP.664 immunized animal, the responding B cell population was initially diverse but underwent marked intra-clonal expansion with repeated immunization. The findings suggest that the expanded clonotypes, three of which produced nAbs, arose mainly after the second SOSIP immunization and persisted for more than 60 weeks. We also present evidence to suggest that the most abundant clonotypes in this animal, neutralizing and non-neutralizing, may have frequently trafficked through LN germinal centers [14]. We are currently investigating whether comparable levels of intra-clonal expansion also occurred in other animals from the same cohort that had lower serum nAb titers.

The potential disconnect between initially primed B cells and those that expanded and persisted was also noted in another HIV vaccine study [18], although it is possible that these observations reflect incomplete sampling [37]. One feature of our study is that we selected B cells directed at BG505 gp120, while excluding those that recognized the gp41 trimer base [10]. In doing so, we successfully identified B cells that produced nAbs with targets that reflected the serum, and similar to our previous studies, these neutralizing clonotypes were among the most abundant [10,18,38]. Of six clonotypes selected for high abundance, three produced nAbs with overlapping epitope footprints exactly in the region that was mapped for serum neutralization [10]. The neutralizing mAbs we isolated used distinct binding modes with dissimilar CDRH3 characteristics and different germline alleles that were commonly found in this animal’s Ag + B cell population. The role of the germline V, D, and J allele usage in the development of nAbs warrants further investigation in animals that developed different levels of nAbs with alternate targets in this BG505 SOSIP.664 immunized cohort.

The best neutralizing mAbs were from clonotype 684, with the shortest CDRH3 of 9 amino acids. However, short CDRH3 lengths, such as 9 amino acids, were not common or enriched in the Ag + B cell population, and were present at a frequency of 2% of all clonotypes (Fig 1E). B cell receptors with such short CDRH3 lengths are also found in humans in both naïve and antigen experienced populations at similar frequencies to what we observed here [19]. The early week 26 antibody (mAb 18) with 98% identity to its V_H_ germline could neutralize as well as later mAbs from other clonotypes with more somatic mutation. Potency also increased within the clonotypes over time. We previously reported similar findings in a different RM HIV vaccine study [18]. Thus, common factors for some vaccine elicited neutralizing clonotypes (or lineages) include mAbs close to germline that can neutralize; epitopes within GHs proximal to the CD4bs; and simultaneous intra-clonal expansion of multiple neutralizing clonotypes. However, in both studies, continued divergence from germline did not consistently lead to increased neutralization potency, which appeared to plateau over time in some instances, or to the acquisition of breadth in any case. It is unlikely that repeated immunizations with the same immunogen, without any targeted modifications, can reliably drive neutralization breadth.

We also determined high resolution antigen-bound structures representing three neutralizing clonotypes. These cryo-EM structures of nAbs elicited by BG505 SOSIP.664 in RM reveal distinct binding modes, navigation of the surrounding glycans to reach the underlying protein surface, variable proximity to the CD4bs, and strain-specific Env contacts, sometimes alongside more conserved elements, that limited neutralization to BG505 (S11 Fig). Our structures revealed that clonotypes 684 and 695 (antibodies 1G3, 1A8, and 4) recognize the 465 GH region with subtly distinct orientations and epitope footprints, while mAb 76 (clonotype 1303) binds with an alternative mode that allows direct engagement of loops D, V5 and the CD4 binding loop. Despite partial overlap with the CD4bs, mAb 76 remains BG505-specific, reflecting dependence on strain-restricted residues and highlighting the challenge of eliciting broadly neutralizing activity (S11 Fig). Given that mAb 76 made direct contact with the CD4 binding loop, we examined sequence variation within the 1303 clonotype (119 paired heavy and light chain sequences). We considered residues in the Env BG505 CD4 binding loop region (I358, R360, F361, A362, N363, S364, S365, G366, G367) that were involved in contact with mAb 76 (E33, Y58, G99, S100, W100A, I100B in heavy; S54, D54A in light). Only position 54 in the light chain showed any variation, with S54G appearing in 11 sequences across time points. This indicates strong conservation of the CDR residues responsible for contacting the CD4 binding loop, rather than ongoing affinity maturation to enhance this engagement. As is evident from the divergence from germline, there are mutations and ‘toggling’ in many other positions. This analysis suggests that recognition of the CD4 binding loop was established early in the 1303 lineage and maintained throughout maturation, while somatic mutation primarily affected other regions of the antibody that may contribute to overall stability, orientation, or contacts outside the CD4 binding loop. Finally, previous cryo-EM polyclonal mapping studies of serum antibodies from BG505 SOSIP.664 immunized RM have also shown that antibodies targeting the 465 GH region display multiple distinct angles of approach and binding modes [15]. That study hypothesized that antibodies that bound to the 465 GH could in some cases sterically interfere with CD4 binding, consistent with our findings.

We have demonstrated that multiple expanded B cell clonotypes simultaneously targeted proximal epitopes in or near the 465 GH, with the antibodies showing both neutralizing and non-neutralizing phenotypes. For two of the three expanded non-neutralizers, the mAbs we tested bound to BG505 gp120 and the SOSIP.664 trimer, did not block VRC01 or CD4-Ig binding, but did compete against the autologous neutralizers. These antibodies likely fail to neutralize because their epitope is not present or accessible on the virion-associated functional Env trimer [15,39]. The three neutralizers bound to BG505 gp120 and the soluble SOSIP.664 trimer and are by definition able to bind to the virion associated functional Env trimer [39]. These antibodies exhibited different capacities to compete with VRC01, CH103, and CD4-Ig that was consistent with the degree of epitope overlap with elements of the CD4bs observed in the bound structures. Taken together, the data suggests that 465 GH neutralizers utilized multiple mechanisms to inhibit virus, that could include (i) obstructing Env-CD4 engagement (mAb 76), (ii) sterically interfering with proximal CD4bs access (mAb 1A8), and (iii) using an undefined CD4-independent mechanism such as slowing entry of adsorbed virus or inter-trimer crosslinking (mAb 4). The findings highlight the complexity of antibody-GH recognition and support that strategies to refine trimer immunogen antigenicity is necessary to eliminate recognition of non-conserved residues that restrict neutralization breadth. The findings also suggest that multiple expanded clonotypes may be necessary to drive up serum neutralization potency but that CD4bs proximal GH antibodies could interfere with eliciting true CD4bs nAbs with the potential for breadth. Strategies are needed to improve the efficiency of repeated immunizations to drive the intra-clonal expansion, recall, and mutation, that appears to be associated with high titer, durable neutralization responses while incorporating more precise epitope targeting and elements of viral diversity. The strain specific epitopes we identified are proximal to the CD4bs, a bnAb target, and even include a conserved CD4bs element in the case of mAb 76. It is possible that the neutralizing clonotypes we characterized here could have acquired breadth if they were exposed to other trimers besides BG505 or immunogens with missing or rearranged glycans, strategies employed by others to drive heterologous neutralization breadth [36,40–45].

## Materials and methods

### Ethics statement

For the previous study, animals were housed at the Emory National Primate Research Center and cared for under guidelines established by the Animal Welfare Act and the NIH Guide for the Care and Use of Laboratory Animals using procedures approved by the Institutional Animal Care and Use Committee of Emory University.

### Study design

The goals of this study were to (i) explore the development of a robust serum nAb response elicited by BG505 SOSIP.664 immunization in a RM at the single Ag + B cell level, (ii) isolate mAbs and characterize their antigen binding and neutralization capacity, and (iii) define neutralization epitopes and interaction with glycan moieties at high resolution. We utilized cryopreserved longitudinal PBMC samples and one LN sample collected from a previous study to carry out 10X Genomics VDJ sequencing and bioinformatic analyses, cloning and expression of recombinant B cell receptor variable domains to produce mAbs, evaluation of mAb functions, cryo-EM, and SPR to provide new insight into how nAbs develop in response to repeated BG505 SOSIP.664 immunization.

### Rhesus macaque immunizations

Complete details of the vaccines, schedule, and challenges have been reported [5] and are summarized in (S1 Table). Here we utilized cryopreserved samples from one animal.

### Antigen specific B cell sorting

Peripheral blood mononuclear cells (PBMC) and lymph node (LN) fine needle aspirates (FNA) were obtained as described in [16,46]. Cryopreserved PBMCs and LN FNAs were processed in small batches for Fluorescence-Activated Cell Sorting (FACS) of Ag + IgG + B cells like that described previously [18]. BG505 gp120-His protein, with or without the N332 glycan, was used to sort Ag + B cells on a Becton Dickinson FACS Aria-III or Symphony S6. Briefly, samples were thawed, added to prewarmed RPMI-1640 media (Cytiva) with 10% FBS (Hyclone, Cytiva), pelleted, washed in cold PBS (Cytiva) with 5% FBS, and adjusted to 1 x 10^5^ cells per μL. Cells were kept on ice and shielded from light at all steps. Cells were incubated with probe at 1 μg per 10^6^ cells for 15 min; then 2 μL of anti-human IgG FITC, 1 μL per 10^6^ cells each of anti-human CD14 PE-Cy7, anti-human CD3 Pacific Blue, anti-human CD20 BV650, anti-His PE, anti-His APC, 0.5 μg of hashing antibody per 10^6^ cells were added. After 30 min, cells were washed with cold PBS + 5% FBS before adding live/dead aqua cell viability dye. After final 30 min incubation and washing, cells were resuspended in ~400 μL RPMI with 10% FBS for sorting. The probes, antibody staining panel, and hashtags are described in S2 Table, with a representative gating strategy in S3 Table. Two samples, week 83 and 93, had inadvertent changes made in the live/dead staining protocol.

### 10X Genomics BCR sequencing

Immediately following sorting, Ag + B cells were encapsulated into Gel Bead-in-Emulsions (GEMs) using the 10X Genomics Chromium iX Controller according to 10X Genomics protocols in the NextGem or GEM-X Single Cell 5’ Reagents Kit with Feature Barcode Technology as described previously [18]. For VDJ libraries, cDNA was amplified using in-house RM immunoglobulin primers [18]. The VDJ, GEX, and CSP libraries were each uniquely indexed: Dual Index Plate TT Set A for VDJ and GEX libraries, and Dual Index Plate TN Set A for CSP libraries, to allow for demultiplexing post-sequencing. All libraries were quantified and assessed for fragment size distribution using the Agilent 4150 TapeStation System with the High Sensitivity D5000 ScreenTape Kit. Final libraries were quantified with an Invitrogen Qubit 4 using HS dsDNA reagents, pooled, and sequenced by Illumina NGS with either NovaSeq 6000 or NextSeq flow cells.

### Generation of mAb expressing plasmid constructs

Selected VDJ regions of Ag + B cells were synthesized as DNA fragments, digested with restriction enzymes, and purified using the QIAquick Gel Extraction Kit (Qiagen). Inserts were ligated into plasmid expression vectors containing human constant regions for IgG1, Igκ, and Igλ [47]. Plasmids were transformed and then purified using QIAprep Spin Miniprep Kit or Plasmid Maxi Kit (Qiagen) and sequence verified. V_H_ and V_L_ expression plasmids for the mAbs 1A8 and 1G3 were synthesized by Sino Biological using VDJ sequences obtained previously [10].

### Production of monoclonal antibodies and CD4-Ig

mAbs from VDJ sequences, HIV-1 bnAbs PGT121, PGT145, CH103, and N49P9.6-FR3, and CD4-Ig (BEI Resources, ARP-12960) were expressed in Expi293F cells (Gibco) as described previously [10,18,29,38]. Cultures were grown at 37 °C with 8% CO₂ with orbital shaking and harvested at 5–7 days post-transfection. mAbs were purified from supernatants using Cytiva Protein G Ab SpinTrap, HiTrap Protein A HP, or Gravitrap columns. bnAbs were further purified by size exclusion chromatography (SEC) on a Cytiva Superdex 200 Increase 10/300 GL column equilibrated in PBS pH 7.4. Purified mAbs were concentrated and frozen for future use.

### Antibody Fab preparation

Antibody antigen-binding fragments (Fabs) were generated by enzymatic digestion of purified mAbs using Thermo Scientific immobilized papain. Fabs were purified by passage over a HiTrap Protein A affinity column followed by SEC purification on a Cytiva Superdex 200 Increase 10/300 GL column equilibrated in PBS. Purified Fab fractions were collected and concentrated for complex formation.

### BG505 SOSIP.664 expression and purification

BG505 SOSIP.664 with (for SPR studies) or without a His tag were expressed in Expi or HEK293 GnTI⁻ cells by transient expression and purified as previously described [29]. Briefly, cells with viability of >90% were co-transfected with plasmids encoding BG505 SOSIP.664 and furin at a molar ratio of 4:1 and incubated for seven days at 37 °C with 8% CO₂ and 125 rpm agitation. BG505 SOSIP.664 was then purified from filtered media using PGT145 affinity chromatography. Bound trimer was eluted with 3 M magnesium chloride and immediately diluted into PBS buffer containing 20 mM Tris-HCl (pH 8.0). Elution fractions were further purified by SEC using a Cytiva Superdex 200 Increase 10/300 GL column pre-equilibrated with PBS and then concentrated for biophysical experiments.

### Biolayer interferometry (BLI)

BLI was performed using the GatorBio Gator Plus Advanced BLI System to assess mAb binding to BG505 gp120 and BG505 SOSIP.664. mAbs were loaded at 10 μg/mL onto anti-human IgG Fc Gen II biosensors to a threshold of 1 nm. The mAb-loaded sensors were introduced into 7 serial 1:1 dilutions of BG505.T332.W6M.C1 gp120-His protein or BG505.T332N.W6M.C1 gp120-His protein (117 nM – 1.8 nM) or BG505 SOSIP.664 (100 nM – 1.5 nM) and one reference well with only K buffer for a 300s association step, followed by a 600s dissociation step. Assays were performed at 30 °C with 400 rpm for loading and 1000 rpm for association and dissociation. Regeneration and neutralization steps were included between cycles. CD4bs bnAb VRC01 was used as a positive control, and EM4C04 mAb (a human antibody specific for 2009 pH1N1 virus hemagglutinin) [48] served as a negative control and double reference subtraction. For BLI competition experiments, the BG505 gp120-His antigen was loaded at 10 μg/mL onto Ni-NTA sensors to a threshold of 0.5 nm. Antigen-loaded sensors were incubated with primary antibody or human recombinant CD4-Ig protein at 30 μg/mL with 400 rpm for 1200s then 30 μg/mL secondary antibody with 400 rpm for 600s. A no primary antibody control was included to determine the maximum shift for each secondary antibody or CD4-Ig. BG505 gp120-His proteins were obtained from commercial vendors while BG505 SOSIP.664 used in these experiments was generated as described previously [38]. Competition assays were performed in both directions, reversing the primary and secondary mAbs, and the residual binding was averaged.

### TZM-bl neutralization assay

HIV-1 Env pseudoviruses were produced as previously described [10,18,38]. Neutralization activity was assessed using the TZM-bl assay as previously described [10,18,38]. In brief, 2000 infectious units of each Env pseudovirus was mixed with mAb at different concentrations and added to 96-well plates containing a TZM-bl monolayer. After 48 hr at 37 °C, cells were lysed, and luciferase activity was measured. Assays were performed in duplicate and independently repeated at least once. IC_50_ values were calculated in GraphPad Prism v10.

### Cryo-EM sample preparation and data collection

BG505 SOSIP.664 was first incubated with a threefold molar excess of PGT121 Fab overnight at 4 °C, purified, and then incubated with a threefold molar excess of Fab from the mAb of interest (e.g., 1G3, 1A8, 4, or 76) and purified by SEC. Peaks corresponding to the individual complexes were collected and concentrated to ~1.4 to ~3 mg/mL.

3 μL of protein complex was deposited on a carbon film copper grid (QUANTIFOIL R 2/1, UT, 300 mesh, EMS cat# Q250-CR1.3-2NM) that had been glow-discharged for 12s at 25 mA using PELCO easiGlow (TedPella Inc.). Cryo-EM grids were prepared using Thermo Scientific Vitrobot Mark IV with a blot time of 3s and variable blot forces at 18 °C and 100% humidity. Cryo-grids were loaded onto an electron microscope and data collected at 200 kV with GATAN K3 (6k x 4k) detectors. Micrographs were collected at magnifications and pixel sizes in S4 Table with a total exposure dose of 46–60 e − /Å^2^ using SerialEM software.

### Cryo-EM data processing, model building and refinement

A total of 2,756 (1G3 Fab-PGT 121 Fab-BG505 SOSIP.664 complex), 977 (1A8 Fab-BG505 SOSIP.664 complex), 7,620 (4 Fab-PGT121 Fab-BG505 SOSIP.664 complex) and 6,849 (76 Fab-PGT121 Fab-BG505 SOSIP.664 complex) movies were collected and motion-corrected using CryoSPARC (Structura Biotechnology Inc., Toronto, CA). All data processing steps – including motion correction, CTF estimation, and manual curation – were carried out in CryoSPARC. Particle picking was performed using the Template Picker with a 400 Å particle diameter. All refinements were performed in C1 (76 Fab) or C3 (1G3, 1A8 and 4 Fabs) symmetry. The 76Fab-PGT121 Fab-BG505 SOSIP.664 complex particles were extracted from both a holey carbon grid (Q250-CR1.3) dataset and a holey carbon grid with ultrathin carbon (Q250-CR1.3-2NM) dataset. Initial model building was using template PDB ID 7UOJ. Initial model-to-map fitting cross-correlation was performed using UCSF ChimeraX [49]. Iterative rounds of model refinement were conducted with Phenix [50] and Coot [51]. Model validations were carried out using EM-Ringer and MolProbity [52]. The final models achieved acceptable geometries, with a Chimera CC scores in the range of 0.75 to 0.82. Data collection, reconstruction, and refinement statistics are provided in S4 Table. Molecular graphics were prepared using PyMOL v2.1.1 (PyMOL Molecular Graphics System, Schrödinger, LLC) and UCSF ChimeraX.

### Buried surface area (BSA) calculations

The interface and buried surface area (BSA) calculations were done with the online webtool PISA [30]. Structural coordinates of BG505 SOSIP–antibody complexes were used as input, and individual components (gp120 and antibody chains) were defined according to the deposited structure. Solvent-accessible surface area (SASA) was computed by PISA using a rolling probe method with a probe radius of 1.4 Å, approximating a water molecule. The BSA was determined as the difference between the sum of SASA values of the unbound components and that of the complex. Interface residues were identified based on a reduction in SASA upon complex formation, as reported by PISA. Where applicable, glycan moieties present in the structures were retained during analysis to account for their contribution to the gp120 surface and antibody interactions. The resulting BSA values were used to quantify the extent of antibody–gp120 interactions and to define epitope regions for each antibody.

### Surface plasmon resonance (SPR)

To assess the effect of antigen glycosylation on binding, two forms of BG505 SOSIP.664 were used: wild-type trimers with complex glycans and trimers with glycans removed by endoglycosidase H_f_ (EndoH). Glycan-null variants were generated by BG505 SOSIP.664 grown in HEK293 GnTI⁻ cells, treated overnight with EndoH, and purified to remove residual glycans. Cytiva anti-His mAb (Cytiva His Capure kit) was first immobilized on CM5 sensor chips to the target level of ~8500 RU as per manufacturer’s protocol. Trimers were then captured by injecting as analyte at a concentration of 3.5 μg/mL and a flow rate of 10 μL/min to ligand attachment level (R_L_) of 275–300 RU. Fabs were injected at the indicated concentrations, and association and dissociation phases were monitored at 30 μL/min for 360s and 800s, respectively. After each binding cycle, the surface was regenerated with a 30s pulse of 10 mM glycine-HCl (pH 1.5) at 30 μL/min and the trimer reloaded. Sensorgrams were processed by subtracting control channel and baseline responses. All binding kinetics parameters were determined by fitting the processed data to a 1:1 Langmuir binding model using Cytiva Biacore Insight Software. Experiments were performed in at least three independent replicates.

### Data processing and bioinformatic analysis

Cellranger v9.0.1 was used to align VDJ and gene expression reads to a custom VDJ reference that included V_H_ germline alleles from RUp16 [18,53] and RM genome v10 NCBI annotation version 103. Only VDJ data consisting of paired heavy and light chain sequences was analyzed here. ScRepertoire [17] was used to make clonotype calls using the Levenshtein distance matrix with similarity threshold of 0.85. Sequence percent identity to the germline of clonotypes that appeared repeatedly across time points were calculated using Biostrings R package version 2.76.0. Significant differences in sequence percent identity were calculated between week 20 and each later time point using the Wilcox Rank sum test.

## Supporting information

S1 Fig Serum neutralization ID_50_ titers for BG505 SOSIP.664 immunized rhesus macaque RUp16 against BG505 Env pseudovirus in the TZM-bl assay. Longitudinal serum samples collected from -4 (baseline) to 121 weeks were tested for neutralization activity against BG505 Env pseudovirus. Some of the ID_50_ titers have been published previously [5,10]. **(A)** The IC_50_ titers are plotted on the y axis on a log10 scale for selected time points. The four SOSIP immunizations and the first and second low dose repeat challenge series (10 and 6 challenges, respectively) are indicated and have been described previously in [5]. **(B)** The actual time points and serum ID_50_ titers are shown.
(TIF)

S2 Fig Longitudinally recurring clonotypes. **(A)** 109 clonotypes were detected in at least 3 of 5 immunization phases: Post-SOSIP 1 (weeks 18, 20); Post-SOSIP 2 (weeks 26, 36), Post-SOSIP 3 (weeks 48, 49), Post-SOSIP 4 (weeks 80, 81, 83), Challenge (weeks 87, 93). These clonotypes were used to evaluate divergence from V_H_ germline over time as shown in Fig 1C. The six clonotypes of interest are indicated by arrows (684, 695, 1303, 385, 386, 599) and more detailed information is shown in **(B)**, including V_H_ and V_L_ gene germline call, CDRH3 length, clonotype size, and the time points in which the clonotype was detected.
(TIF)

S3 Fig Binding of representative mAbs from the six clonotypes to BG505 Env gp120 and BG505 SOSIP.664. Representative BLI kinetics traces show the association (0s-300s) and dissociation (300s-900s) phases for each mAb binding to either BG505 gp120 (117nM) or BG505 SOSIP.664 (100nM). The nm shift is shown on the y axis, and the association/dissociation time (transition indicated by a dashed line) is shown in seconds on the x axis. VRC01 was included as a reference and EM4C04 was included as a negative control (no binding was detected for EM4C04). The antibodies were immobilized on anti-human IgG Fc sensors and dipped into solution containing protein at varying concentrations. All antibodies were tested at 10 μg/mL (67nM). Only the trace of the highest concentration of each protein is shown. The clonotypes are indicated and are divided by non-neutralizers **(A)** and neutralizers **(B)**.
(TIF)

S4 Fig Competition matrix for binding to BG505 gp120 using BLI epitope binning. **(A)** Competition is expressed as the percent residual binding of the secondary antibody in the presence of the primary antibody compared to no primary antibody. bnAb VRC01 was used to test for binding proximal to the CD4bs; EM4C04 was used as a negative control that does not bind HIV Env. mAb IDs are indicated along the right and the bottom of the matrix; clonotypes are indicated on the left and top. Residual binding is shown on a color gradient from 0% (red) to 100% (green) with the value indicated in each box. **(B)** Reduction of VRC01 binding by each antibody is depicted by the height and color of each segment, corresponding to **(A)**. Clonotype numbers are shown on the inside; mAbs are indicated on the outside. mAbs 18, 32, 1A8, 1G3 (clonotype 684), 76 (clonotype 1303), and 4, 70, and 12 (clonotype 695) are neutralizers.
(TIF)

S5 Fig Cryo-EM Data processing and resolution assessment of the BG505 SOSIP.664 complex. **(A-D)** Left panels show representative particle images from selected 2D classes used for *ab initio* map reconstruction of mAbs **(A)** 1G3, **(B)** 1A8, **(C)** 4 and **(D)** 76. Right panels show Fourier shell correlation (FSC) curves calculated with a spherical mask indicate the overall map resolution using the 0.143 cutoff criterion as determined by CryoSPARC. **(E)** Local resolution estimation mapped onto the surface of the complex with side views. Resolution estimates are colored from blue (higher resolution) to red (lower resolution), as indicated.
(TIF)

S6 Fig SPR binding profiles for Fabs 1G3, 1A8, 4, 76 and N49P9.6-FR. SPR sensorgrams are shown for **(A)** fully glycosylated BG505 SOSIP.664 trimers and **(B)** deglycosylated BG505 SOSIP.664 trimers. The sensorgrams display the specific binding responses (in response units, RUs, y-axis) as a function of time (x-axis) during the association and dissociation phases across a range of Fab concentrations (15–500 nM). Sensorgrams corresponding to different antibody concentrations are color-coded, while global fits to a 1:1 binding model are represented in black.
(TIF)

S7 Fig Two monoclonal antibodies were also tested against heterologous Env pseudoviruses representing a global reference panel. The MLV Env pseudovirus was used as a negative control; BG505 Env was also included alongside the 12 reference Envs indicated in the legend as a positive control.
(TIF)

S8 Fig Molecular interactome and epitope characterization. **(A)** Comparative interaction network of Fabs 4 and 1A8 with gp120, mapped in a circle using a 4 Å cutoff criterion. V_H_ and V_L_ residues are shown in the outer circle, with gp120 residues displayed inside the grey circle. Dashed blue lines represent the hydrogen bonds**. (B)** Contact residues, defined by a 5 Å cutoff, shown above the mAb V_L_ (upper panel) and V_H_ (lower panel) sequence with (+) for side-chain contacts and (−) for main-chain contacts. Contact types are color-coded: hydrophilic (blue), hydrophobic (green), and mixed (black). CDRs are colored as in **A**.
(TIF)

S9 Fig CDR-guided epitope analysis of mAbs 1G3, 1A8, 4, and 76. **(A)** Zoomed-view of the interface between gp120 and mAb 76 illustrating the interaction between gp120 loop V5 (black) and mAb 76 CDRL3 (brown) and CDRL1 (blue). All interacting residues are shown as sticks with interactions shown as black dotted lines and glycans shown as grey spheres. **(B)** Contact residues, for mAbs 1G3, 1A8, 4 and 76 with bnAb N49P9.6-FR included for comparison, defined by a 5 Å cutoff, indicated above the gp120 sequence with (+) for side-chain contacts and (−) for main-chain contacts. Contact types are color-coded: hydrophilic (blue), hydrophobic (green), and mixed (black). All complexes were determined with BG505 SOSIP.664. The HXB2 sequence is shown on top for reference. Loop D, loop V5 and the CD4 binding loop are colored as labeled.
(TIF)

S10 Fig Buried surface area (BSA) of CD4 binding site antibodies (CD4bs) in complex with SOSIP.664 trimer or gp120. BSA of the Env antigen as a function of residue contributed by mAbs VRC01 (PDB id: 5FYJ), 04_A06 (PDB id: 8ULT), VRC13 (PDB id: 4YDJ), 3BNC117 (PDB id: 5V8M) and CH103 (PDB id: 4JAN) [23,32,33] as calculated by PISA [30] shown as bars. The BSA is divided into separate contributions by V_H_ and V_L_ for protein (shown in darker and lighter shades, respectively), but combined contributions by Fab for glycan (shown in grey).
(TIF)

S11 Fig Sequence conservation within the gp120 epitope of 1G3, 1A8, 4 and 76 as compared to selected CD4 binding site mAbs. A sequence conservation analysis of HIV-1 gp120 was performed using HIV Env sequences from the HIV Sequence Compendium (https://www.hiv.lanl.gov) aligned to the HXB2 reference strain (clade B), which serves as a standardized residue numbering scheme for HIV Env. The molecular surface of gp120 is displayed and color-coded according to sequence conservation. Residues in the alignment that differ from the HXB2 sequence at that position with a low frequency are shown in dark blue, indicating high conservation, whereas residues that differ from HXB2 at high frequency are shown in red, representing highly variable regions, in a range that spans 0.2% to 99.9% with an average of 5343 sequences used to calculate the frequency at any given residue position. Epitopes that are overlaid on the gp120 surface are based upon the epitope determined from the BSA calculations (Figs 6 and S10). Epitope-associated glycans are represented as black sticks and other glycans as grey sticks. The CD4 binding site is demarcated with an arrow and is labeled.
(TIF)

S1 Table Summary of previous vaccine study with time points shown in weeks. Vaccination groups, group size, and the time points for immunizations and challenges are indicated.
(DOCX)

S2 Table Antibody panel for sorting Ag + RM B cells. Reagent, vendor, and catalog numbers are shown for antibodies used for the cell sorting process.
(DOCX)

S3 Table FACSDiva gating strategy shown from representative sample week 26 PBMC. Table shows hierarchical proportions of cell types during flow cytometric sorting of antigen specific B cells.
(DOCX)

S4 Table Cryo-EM data collection and refinement statistics. Table summarizes the data collection, image processing, and model refinement statistics for the structures of the complex of BG505 SOSIP.664, a chaperone PGT121 Fab, and the Fabs of four mAbs isolated from RUp16.
(DOCX)
