# Supplementary figures and images for: HIV-1 BG505 SOSIP immunization induced B cell expansion targeting the 465-glycan hole, with neutralizing antibodies exhibiting distinct binding modes and mechanisms of virus inhibition
Source: PLoS Pathog. 2026 Jun 5;22(6):e1014268. doi: 10.1371/journal.ppat.1014268 (PMC13262937; doi:10.1371/journal.ppat.1014268)

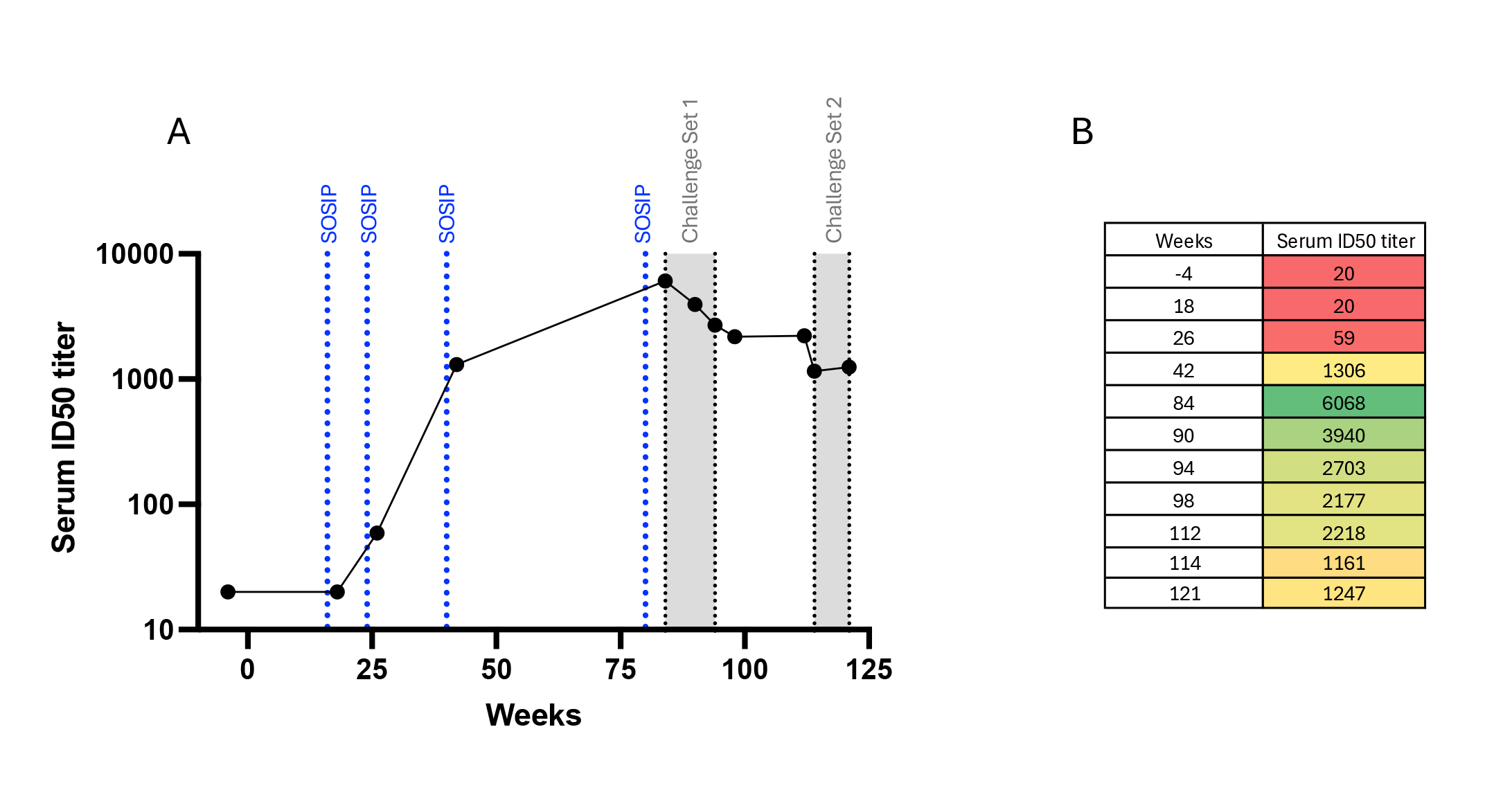

Supplement: S1 Fig — Longitudinal serum samples collected from -4 (baseline) to 121 weeks were tested for neutralization activity against BG505 Env pseudovirus. Some of the ID50 titers have been published previously [5,10]. (A) The IC50 titers are plotted on the y axis on a log10 scale for selected time points. The four SOSIP immunizations and the first and second low dose repeat challenge series (10 and 6 challenges, respectively) are indicated and have been described previously in [5]. (B) The actual time points and serum ID50 titers are shown. (TIF) [file ppat.1014268.s001.tif]

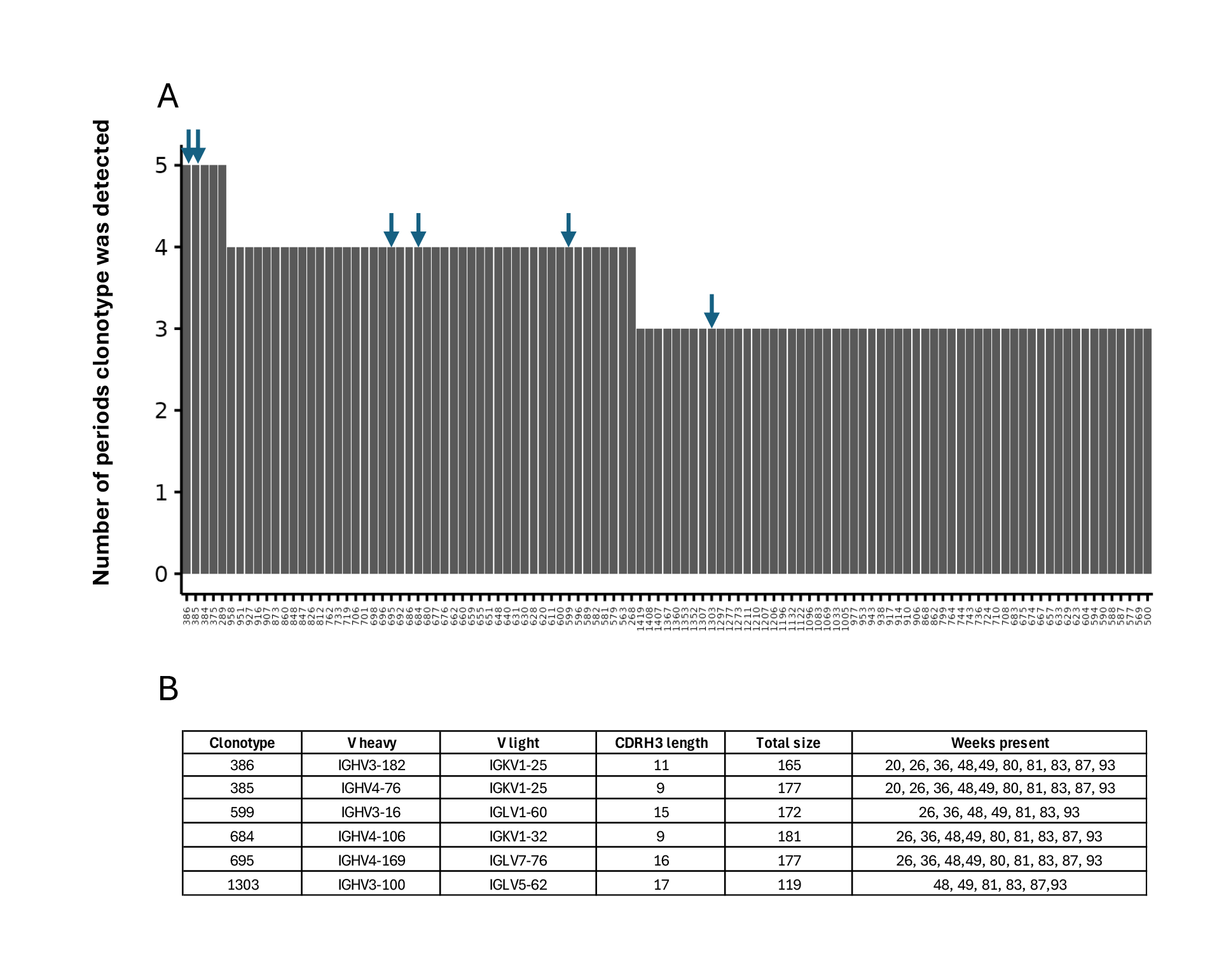

Supplement: S2 Fig — (A) 109 clonotypes were detected in at least 3 of 5 immunization phases: Post-SOSIP 1 (weeks 18, 20); Post-SOSIP 2 (weeks 26, 36), Post-SOSIP 3 (weeks 48, 49), Post-SOSIP 4 (weeks 80, 81, 83), Challenge (weeks 87, 93). These clonotypes were used to evaluate divergence from VH germline over time as shown in Fig 1C. The six clonotypes of interest are indicated by arrows (684, 695, 1303, 385, 386, 599) and more detailed information is shown in (B), including VH and VL gene germline call, CDRH3 length, clonotype size, and the time points in which the clonotype was detected. (TIF) [file ppat.1014268.s002.tif]

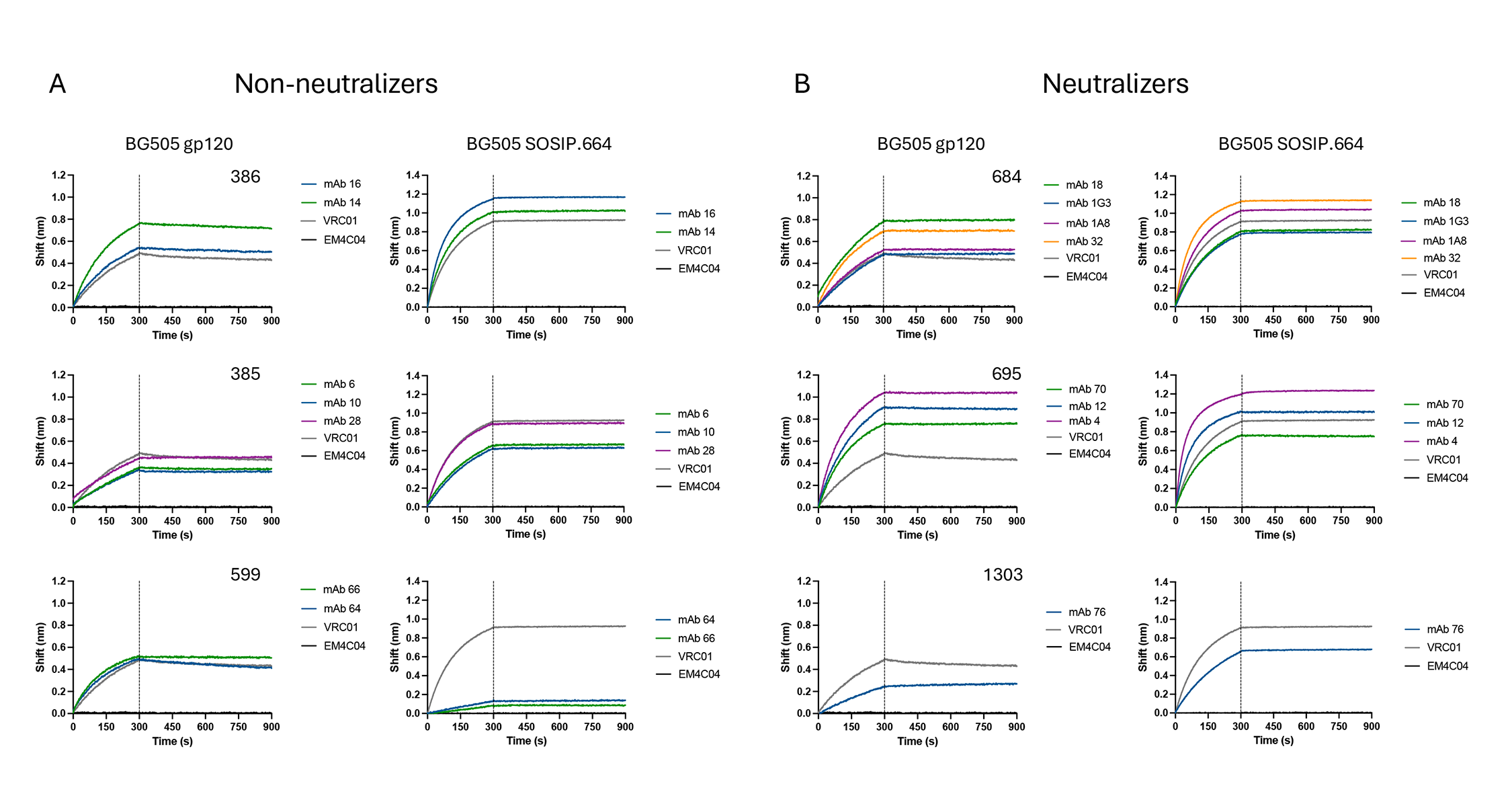

Supplement: S3 Fig — Representative BLI kinetics traces show the association (0s-300s) and dissociation (300s-900s) phases for each mAb binding to either BG505 gp120 (117nM) or BG505 SOSIP.664 (100nM). The nm shift is shown on the y axis, and the association/dissociation time (transition indicated by a dashed line) is shown in seconds on the x axis. VRC01 was included as a reference and EM4C04 was included as a negative control (no binding was detected for EM4C04). The antibodies were immobilized on anti-human IgG Fc sensors and dipped into solution containing protein at varying concentrations. All antibodies were tested at 10 μg/mL (67nM). Only the trace of the highest concentration of each protein is shown. The clonotypes are indicated and are divided by non-neutralizers (A) and neutralizers (B). (TIF) [file ppat.1014268.s003.tif]

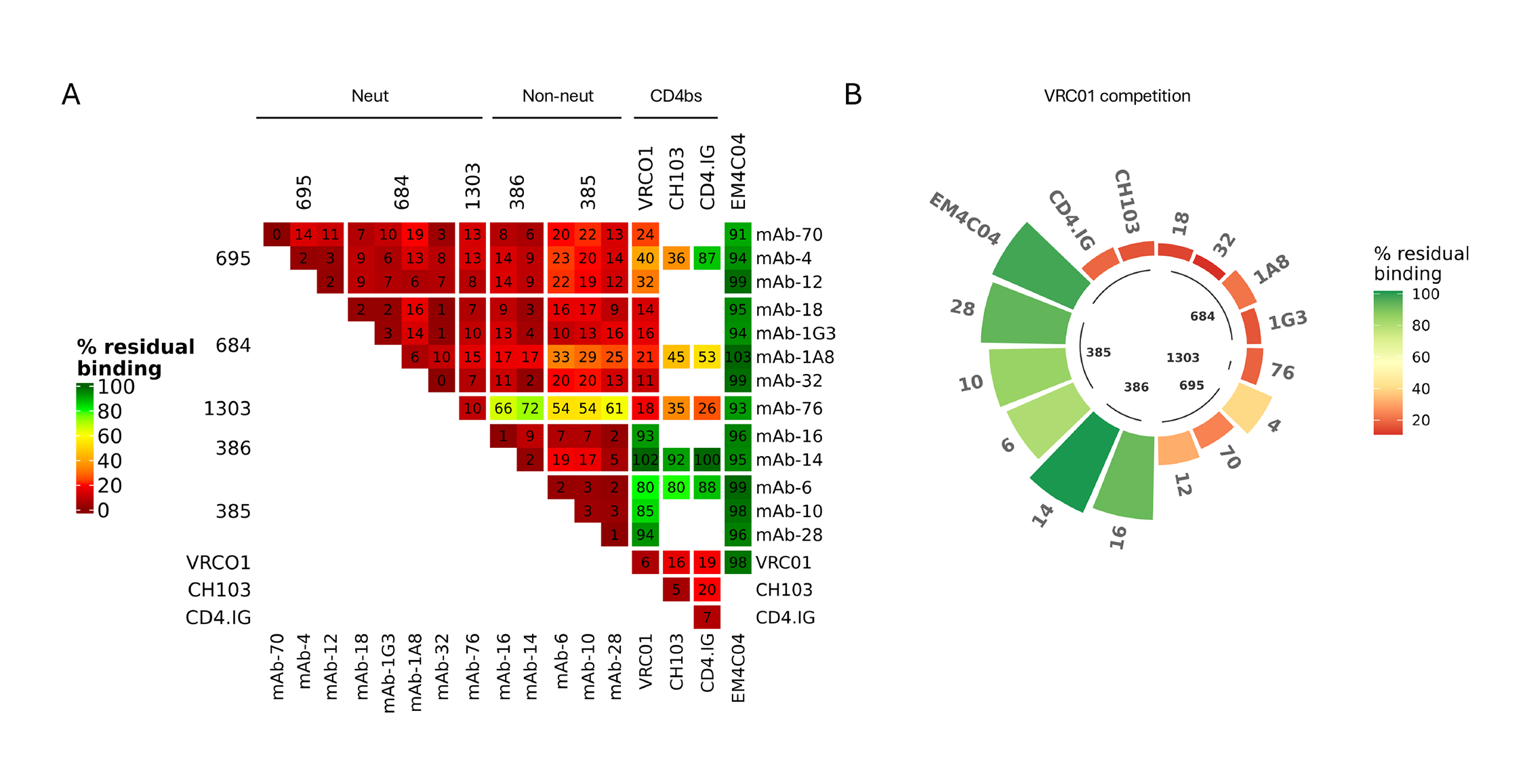

Supplement: S4 Fig — (A) Competition is expressed as the percent residual binding of the secondary antibody in the presence of the primary antibody compared to no primary antibody. bnAb VRC01 was used to test for binding proximal to the CD4bs; EM4C04 was used as a negative control that does not bind HIV Env. mAb IDs are indicated along the right and the bottom of the matrix; clonotypes are indicated on the left and top. Residual binding is shown on a color gradient from 0% (red) to 100% (green) with the value indicated in each box. (B) Reduction of VRC01 binding by each antibody is depicted by the height and color of each segment, corresponding to (A). Clonotype numbers are shown on the inside; mAbs are indicated on the outside. mAbs 18, 32, 1A8, 1G3 (clonotype 684), 76 (clonotype 1303), and 4, 70, and 12 (clonotype 695) are neutralizers. (TIF) [file ppat.1014268.s004.tif]

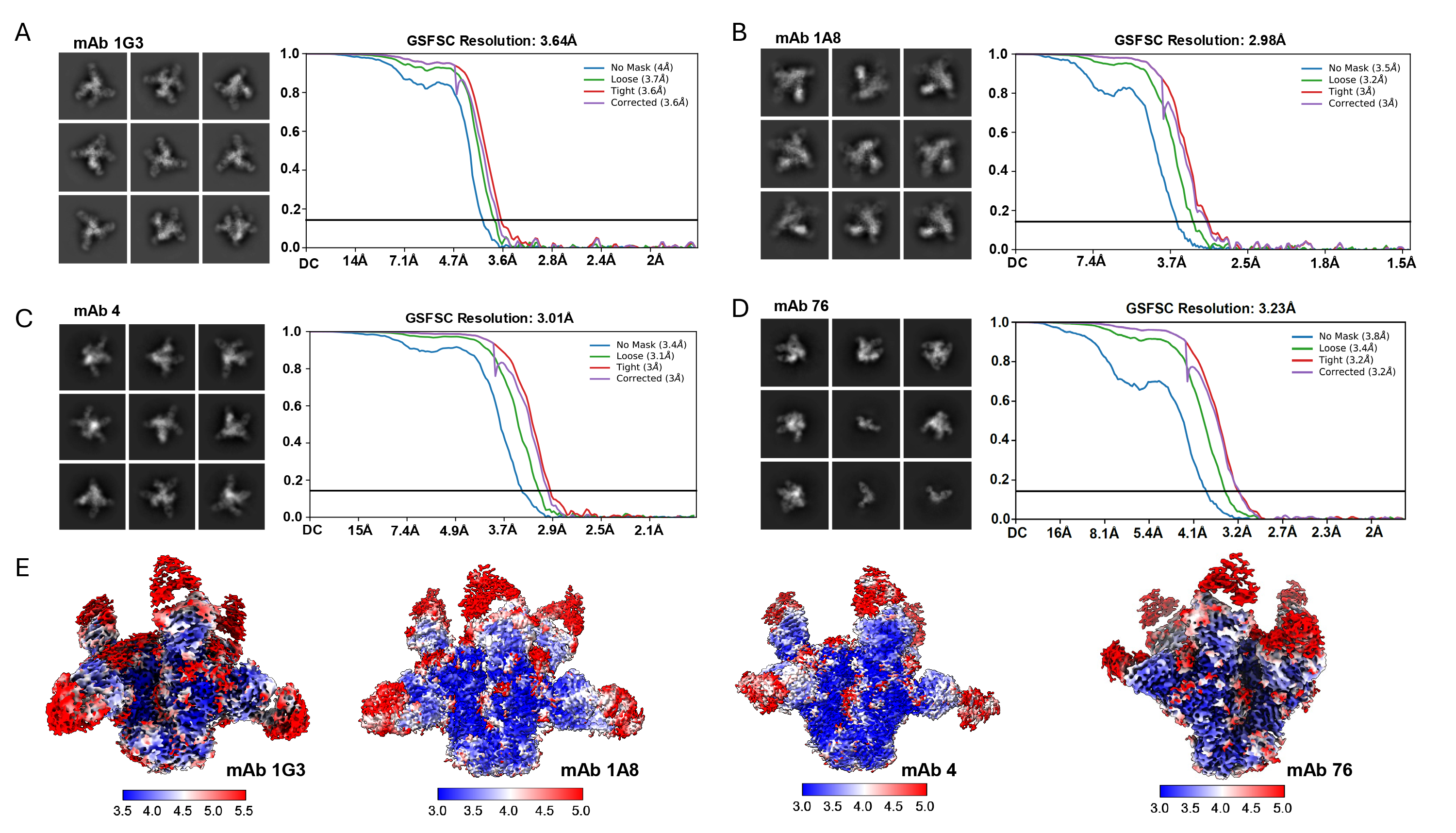

Supplement: S5 Fig — (A-D) Left panels show representative particle images from selected 2D classes used for ab initio map reconstruction of mAbs (A) 1G3, (B) 1A8, (C) 4 and (D) 76. Right panels show Fourier shell correlation (FSC) curves calculated with a spherical mask indicate the overall map resolution using the 0.143 cutoff criterion as determined by CryoSPARC. (E) Local resolution estimation mapped onto the surface of the complex with side views. Resolution estimates are colored from blue (higher resolution) to red (lower resolution), as indicated. (TIF) [file ppat.1014268.s005.tif]

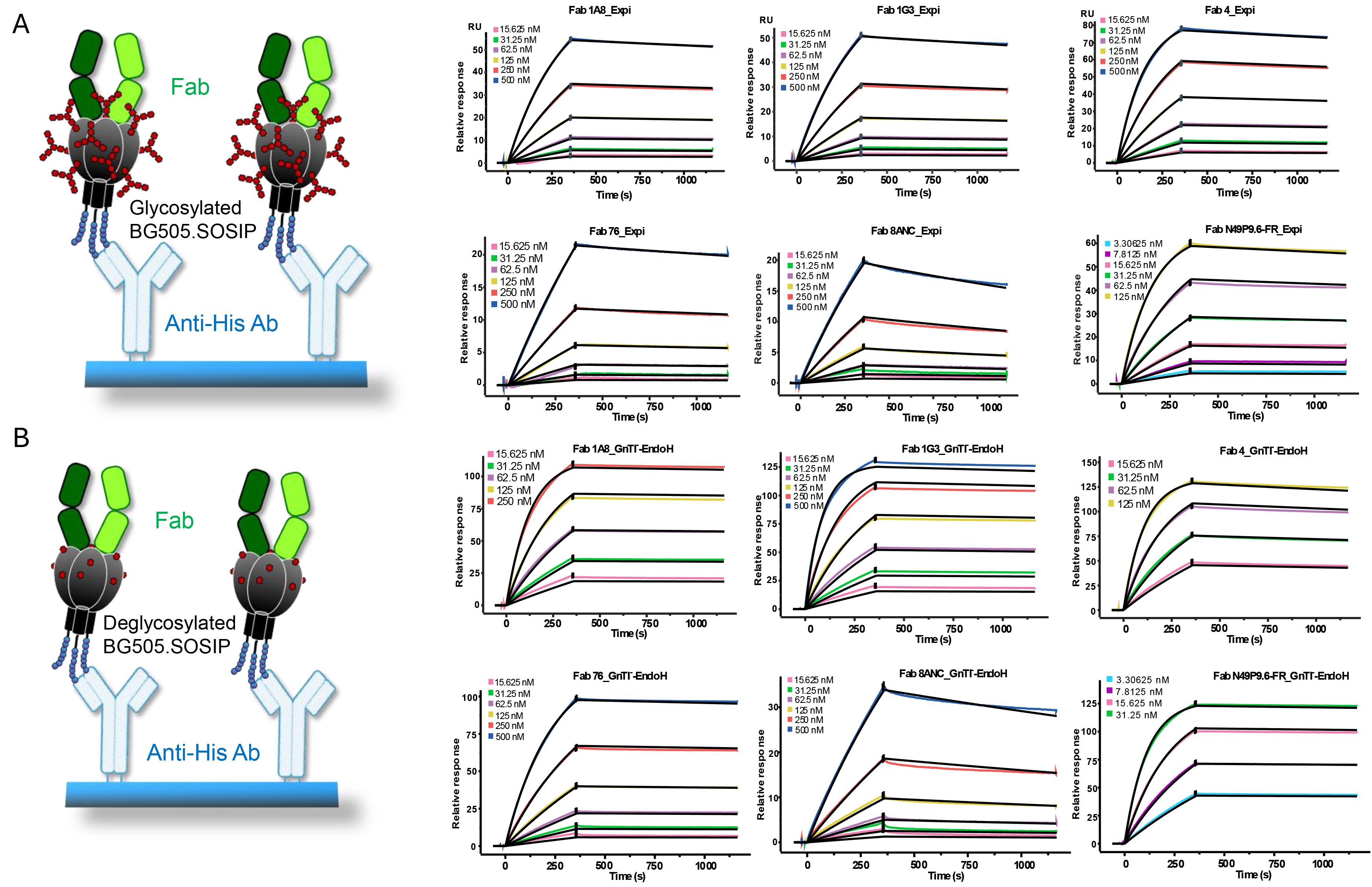

Supplement: S6 Fig — SPR sensorgrams are shown for (A) fully glycosylated BG505 SOSIP.664 trimers and (B) deglycosylated BG505 SOSIP.664 trimers. The sensorgrams display the specific binding responses (in response units, RUs, y-axis) as a function of time (x-axis) during the association and dissociation phases across a range of Fab concentrations (15–500 nM). Sensorgrams corresponding to different antibody concentrations are color-coded, while global fits to a 1:1 binding model are represented in black. (TIF) [file ppat.1014268.s006.tif]

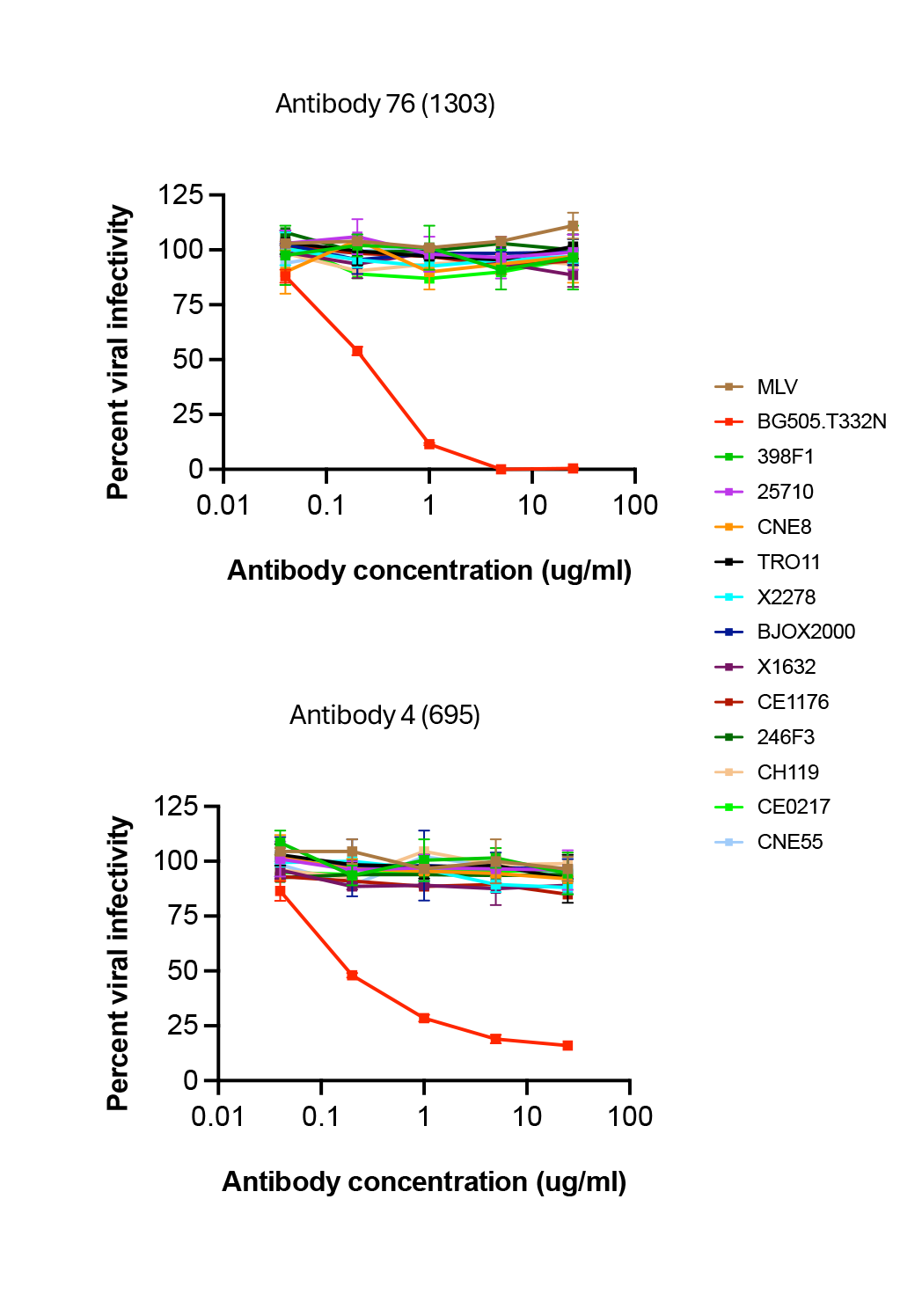

Supplement: S7 Fig — The MLV Env pseudovirus was used as a negative control; BG505 Env was also included alongside the 12 reference Envs indicated in the legend as a positive control. (TIF) [file ppat.1014268.s007.tif]

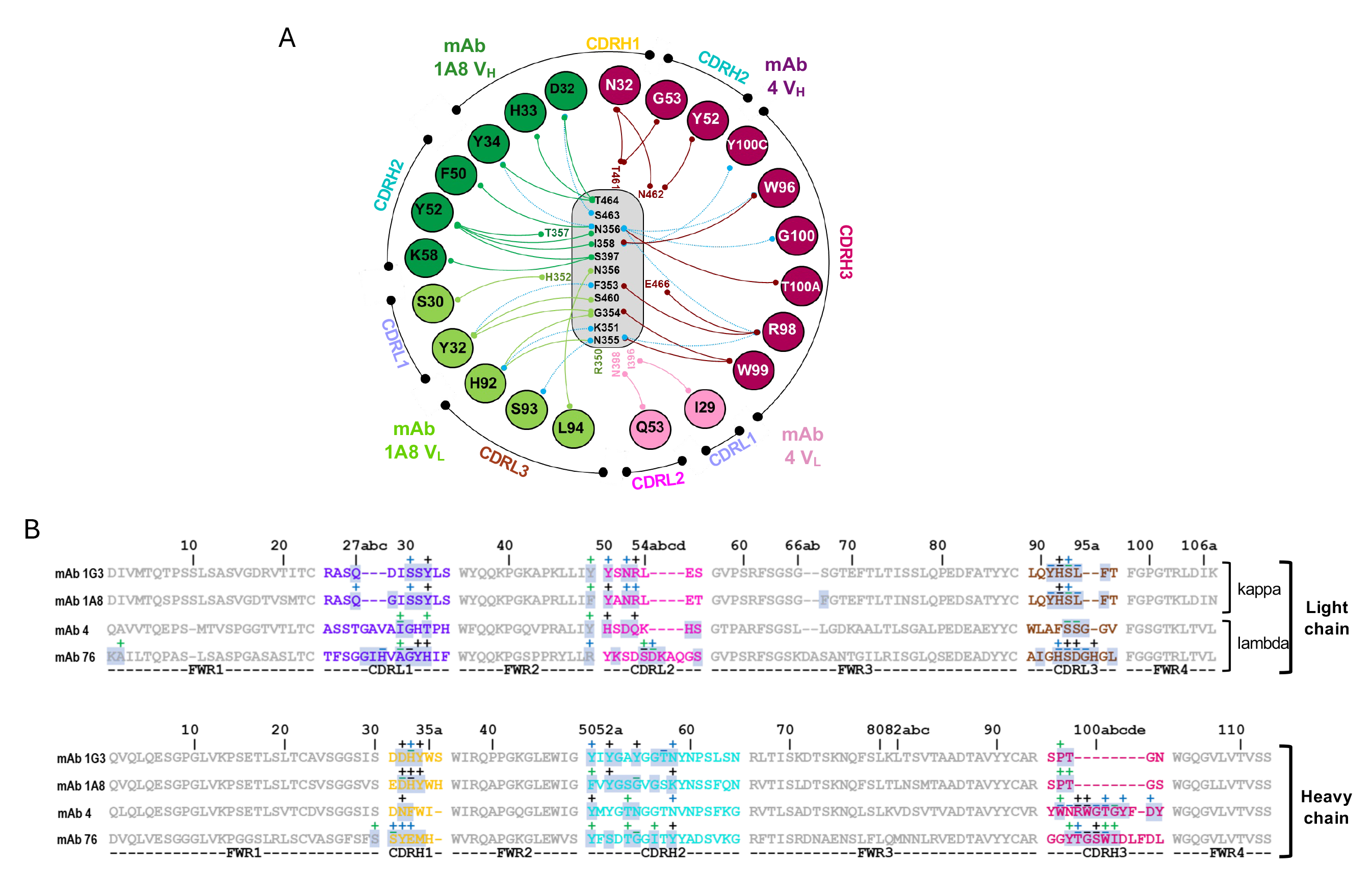

Supplement: S8 Fig — (A) Comparative interaction network of Fabs 4 and 1A8 with gp120, mapped in a circle using a 4 Å cutoff criterion. VH and VL residues are shown in the outer circle, with gp120 residues displayed inside the grey circle. Dashed blue lines represent the hydrogen bonds. (B) Contact residues, defined by a 5 Å cutoff, shown above the mAb VL (upper panel) and VH (lower panel) sequence with (+) for side-chain contacts and (−) for main-chain contacts. Contact types are color-coded: hydrophilic (blue), hydrophobic (green), and mixed (black). CDRs are colored as in A. (TIF) [file ppat.1014268.s008.tif]

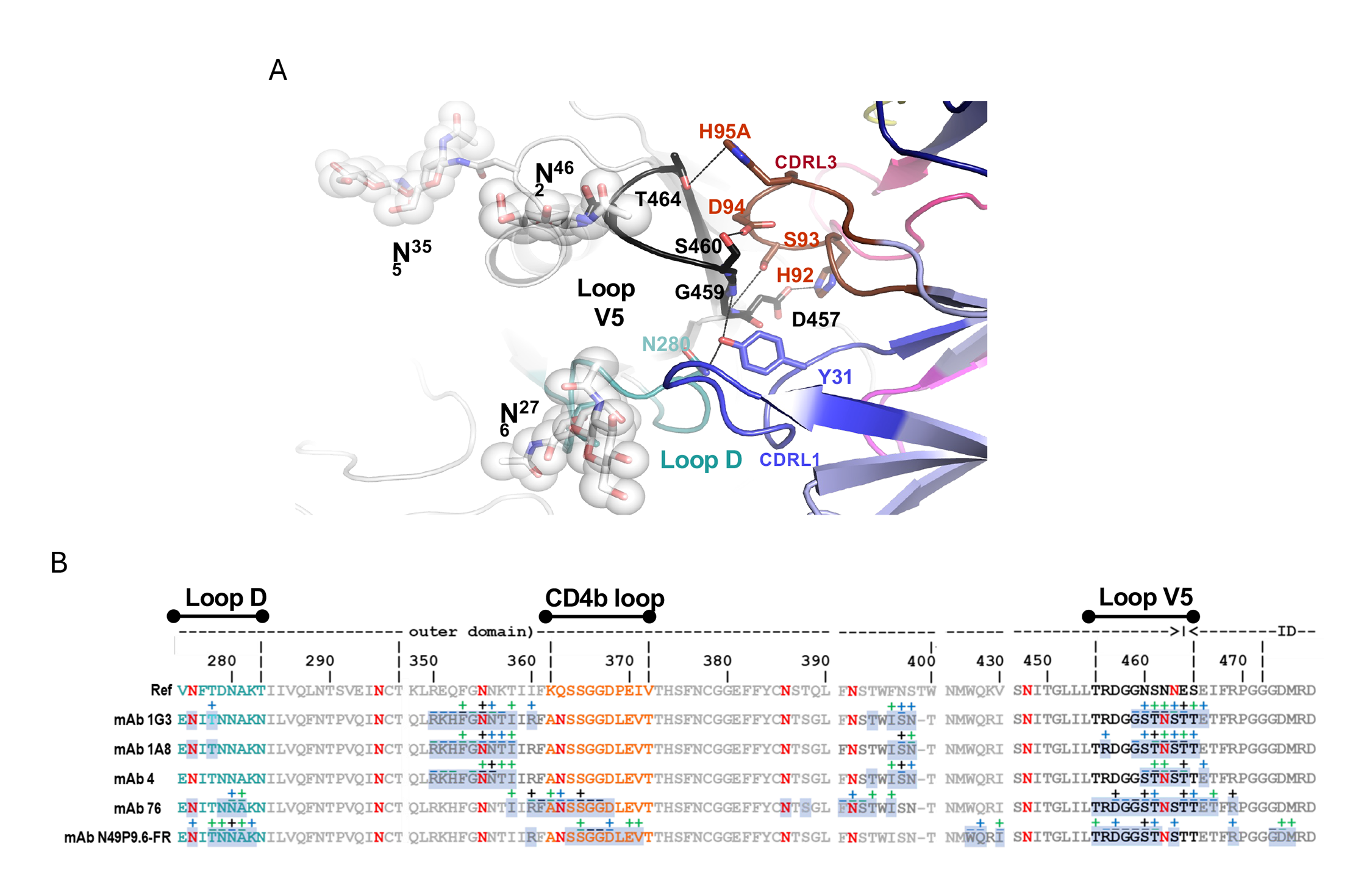

Supplement: S9 Fig — (A) Zoomed-view of the interface between gp120 and mAb 76 illustrating the interaction between gp120 loop V5 (black) and mAb 76 CDRL3 (brown) and CDRL1 (blue). All interacting residues are shown as sticks with interactions shown as black dotted lines and glycans shown as grey spheres. (B) Contact residues, for mAbs 1G3, 1A8, 4 and 76 with bnAb N49P9.6-FR included for comparison, defined by a 5 Å cutoff, indicated above the gp120 sequence with (+) for side-chain contacts and (−) for main-chain contacts. Contact types are color-coded: hydrophilic (blue), hydrophobic (green), and mixed (black). All complexes were determined with BG505 SOSIP.664. The HXB2 sequence is shown on top for reference. Loop D, loop V5 and the CD4 binding loop are colored as labeled. (TIF) [file ppat.1014268.s009.tif]

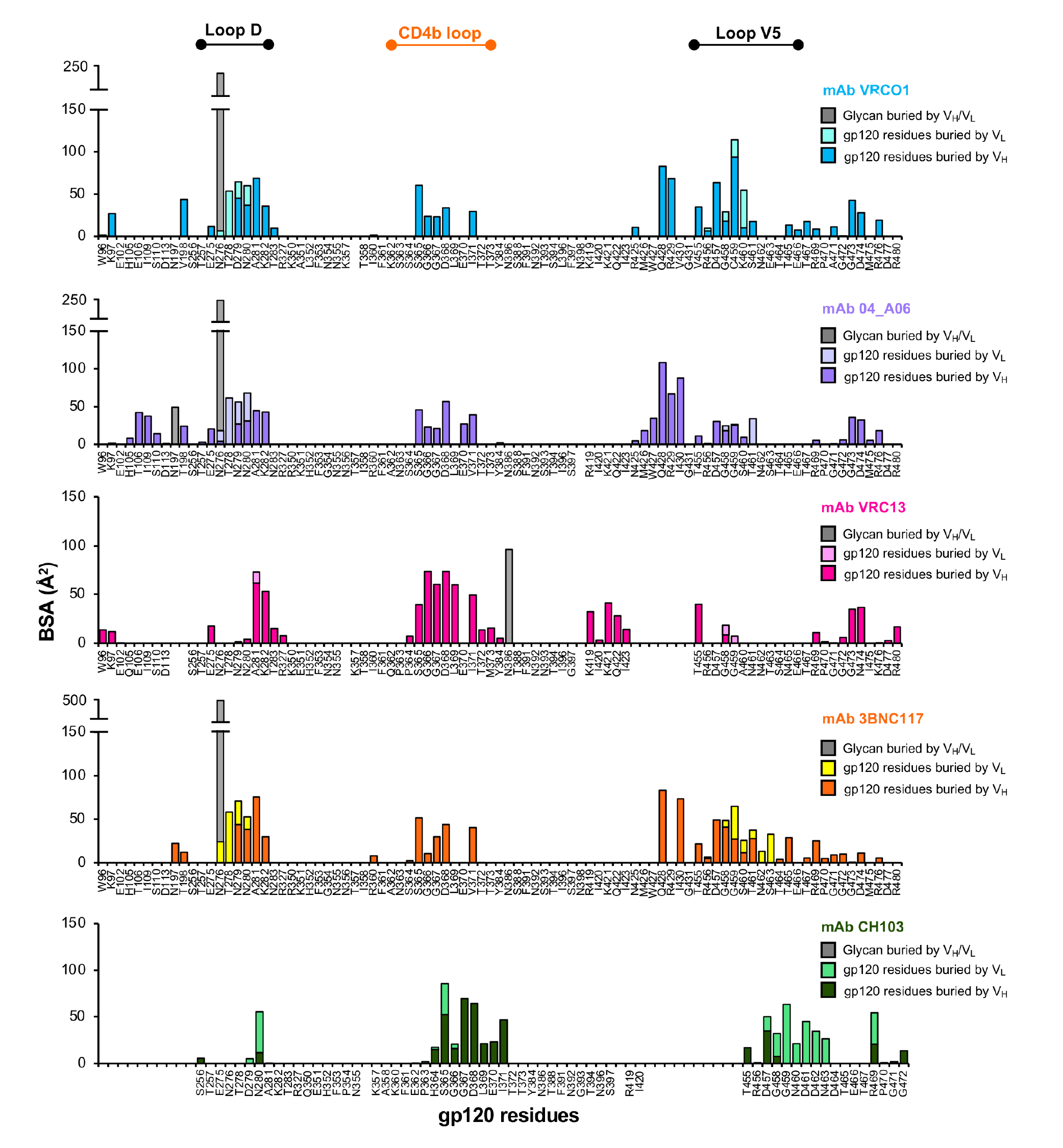

Supplement: S10 Fig — BSA of the Env antigen as a function of residue contributed by mAbs VRC01 (PDB id: 5FYJ), 04_A06 (PDB id: 8ULT), VRC13 (PDB id: 4YDJ), 3BNC117 (PDB id: 5V8M) and CH103 (PDB id: 4JAN) [23,32,33] as calculated by PISA [30] shown as bars. The BSA is divided into separate contributions by VH and VL for protein (shown in darker and lighter shades, respectively), but combined contributions by Fab for glycan (shown in grey). (TIF) [file ppat.1014268.s010.tif]

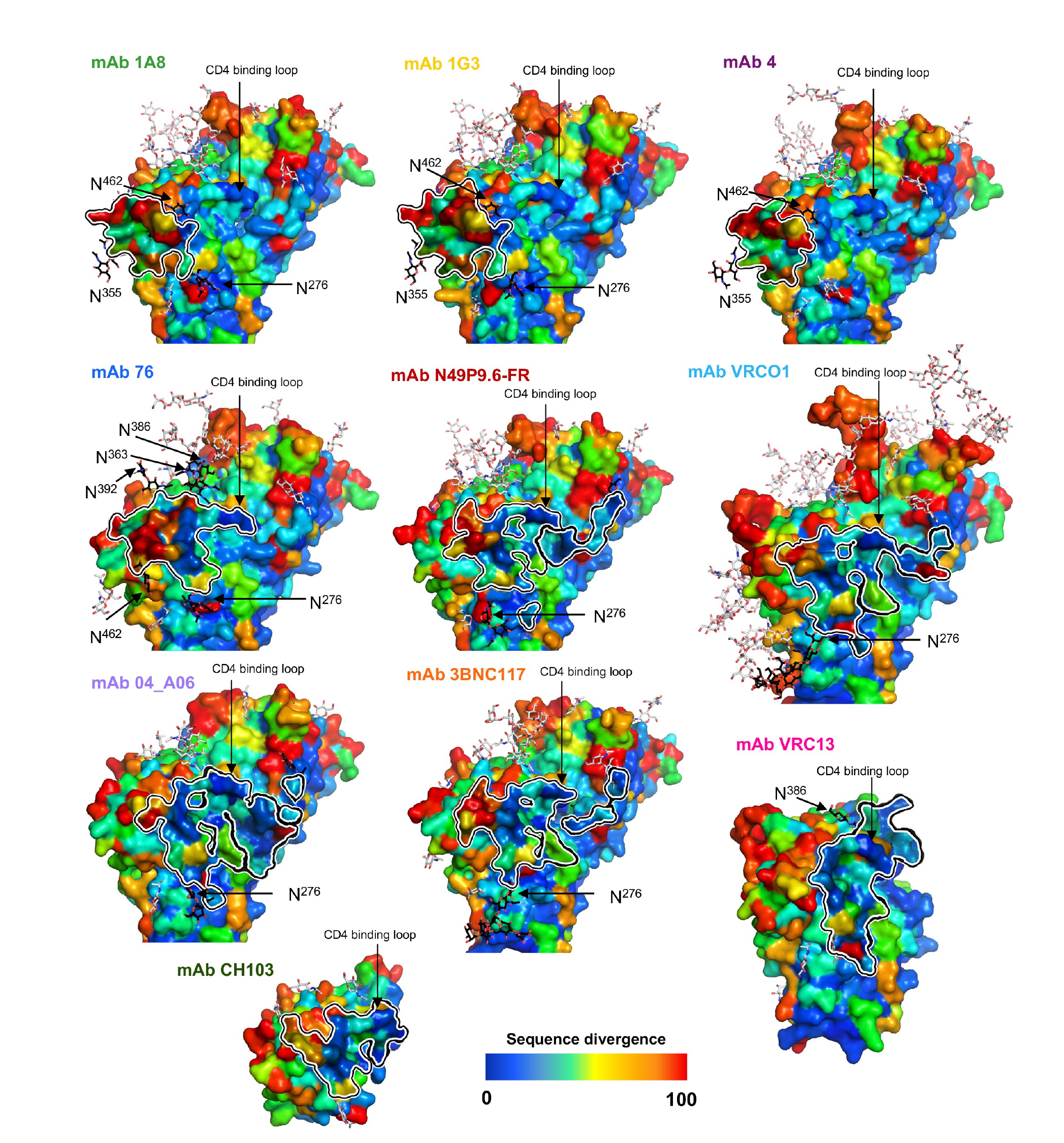

Supplement: S11 Fig — A sequence conservation analysis of HIV-1 gp120 was performed using HIV Env sequences from the HIV Sequence Compendium (https://www.hiv.lanl.gov) aligned to the HXB2 reference strain (clade B), which serves as a standardized residue numbering scheme for HIV Env. The molecular surface of gp120 is displayed and color-coded according to sequence conservation. Residues in the alignment that differ from the HXB2 sequence at that position with a low frequency are shown in dark blue, indicating high conservation, whereas residues that differ from HXB2 at high frequency are shown in red, representing highly variable regions, in a range that spans 0.2% to 99.9% with an average of 5343 sequences used to calculate the frequency at any given residue position. Epitopes that are overlaid on the gp120 surface are based upon the epitope determined from the BSA calculations (Figs 6 and S10). Epitope-associated glycans are represented as black sticks and other glycans as grey sticks. The CD4 binding site is demarcated with an arrow and is labeled. (TIF) [file ppat.1014268.s011.tif]
