# Supplementary material for: HIV-1 BG505 SOSIP immunization induced B cell expansion targeting the 465-glycan hole, with neutralizing antibodies exhibiting distinct binding modes and mechanisms of virus inhibition
Source: PLoS Pathog. 2026 Jun 5;22(6):e1014268. doi: 10.1371/journal.ppat.1014268 (PMC13262937; doi:10.1371/journal.ppat.1014268)
Supplement: S1 Table — Vaccination groups, group size, and the time points for immunizations and challenges are indicated. (DOCX) [file ppat.1014268.s012.docx]

**S1 Table. Summary of previous vaccine study with time points shown in weeks.**

| **Vaccine Group** | **VSV-Gag** | **VV-Gag** | **BG505**  **SOSIP** | **BG505**  **SOSIP** | **Ad5-Gag** | **BG505**  **SOSIP** | **BG505**  **SOSIP** | **SHIV**  **Challenge 1** | **SHIV**  **Challenge**  **2** |
| --- | --- | --- | --- | --- | --- | --- | --- | --- | --- |
| SOSIP/3M-052 (n=15) |  |  | 16 | 24 |  | 40 | 80 | 84-94 | 114-121 |
| HVV + SOSIP/3M-052 (n=15) | 0 | 8 | 16 | 24 | 36 | 40 | 80 | 84-94 | 114-121 |

Vaccination groups, group size, and the time points for immunizations and challenges are indicated.
