# Supplementary material for: HIV-1 BG505 SOSIP immunization induced B cell expansion targeting the 465-glycan hole, with neutralizing antibodies exhibiting distinct binding modes and mechanisms of virus inhibition
Source: PLoS Pathog. 2026 Jun 5;22(6):e1014268. doi: 10.1371/journal.ppat.1014268 (PMC13262937; doi:10.1371/journal.ppat.1014268)
Supplement: S2 Table — Reagent, vendor, and catalog numbers are shown for antibodies used for the cell sorting process. (DOCX) [file ppat.1014268.s013.docx]

**S2 Table. Antibody panel for sorting Ag+ RM B cells.**

| **Reagent** | **Supplier** | **Catalog Number** |
| --- | --- | --- |
| TotalSeq-C Hashing Antibody #1 | Biolegend | 394661 |
| TotalSeq-C Hashing Antibody #2 | Biolegend | 394663 |
| TotalSeq-C Hashing Antibody #3 | Biolegend | 394665 |
| TotalSeq-C Hashing Antibody #4 | Biolegend | 394667 |
| TotalSeq-C Hashing Antibody #5 | Biolegend | 394669 |
| Ultracomp eBeads Compensation beads | Invitrogen | 01-3333-42 |
| Antigen probe (BG505.T332.W6M.C1 gp120-His) | ImmuneTech | IT-001-176p |
| Antigen probe (BG505.T332N.W6M.C1 gp120-His) | Thermo Fisher | custom project (23AD433C_3686362) |
| LIVE/DEAD Fixable Aqua Dead Cell Stain Kit | Life technologies | L34966 |
| Pacific Blue anti-Human CD3, clone SP34-2 IgG1 lambda | Becton Dickinson | 558124 |
| PE-Cy 7 anti-Human CD14, clone M5E2 | Becton Dickinson | 557742 |
| Brilliant violet 650 anti-Human CD20, clone 2h7 | Biolegend | 302366 |
| FITC anti-Human IgG, clone G18-145 | Becton Dickinson | 555786 |
| PE anti-His, clone GG11-8F3.5.1 | Miltenyi Biotec | 130-120-718 |
| APC anti-His, clone GG11-8F3.5.1 | Miltenyi Biotec | 130-119-782 |

Reagent, vendor, and catalog numbers are shown for antibodies used for the cell sorting process.
