# Supplementary material for: HIV-1 BG505 SOSIP immunization induced B cell expansion targeting the 465-glycan hole, with neutralizing antibodies exhibiting distinct binding modes and mechanisms of virus inhibition
Source: PLoS Pathog. 2026 Jun 5;22(6):e1014268. doi: 10.1371/journal.ppat.1014268 (PMC13262937; doi:10.1371/journal.ppat.1014268)
Supplement: S3 Table — Table shows hierarchical proportions of cell types during flow cytometric sorting of antigen specific B cells. (DOCX) [file ppat.1014268.s014.docx]

**S3 Table. FACSDiva gating strategy shown from representative sample week 26 PBMC.**

| **Population** | **# Events** | **% Parent** | **% Total** |
| --- | --- | --- | --- |
| All | 4,484,772 | 100.0 | 100.0 |
| Lymphocytes | 3,961,709 | 88.3 | 88.3 |
| Singlets | 3,811,952 | 96.2 | 85.0 |
| Live | 3,352,519 | 87.9 | 74.8 |
| CD14- | 3,268,376 | 97.5 | 72.9 |
| CD3- | 1,308,645 | 40.0 | 29.2 |
| CD20+ | 832,416 | 63.6 | 18.6 |
| IgG+ | 239,514 | 28.8 | 5.3 |
| Double His+ Ag+ | 3,575 | 1.5 | 0.1 |

Table shows hierarchical proportions of cell types during flow cytometric sorting of antigen specific B cells.
