# Supplementary material for: HIV-1 BG505 SOSIP immunization induced B cell expansion targeting the 465-glycan hole, with neutralizing antibodies exhibiting distinct binding modes and mechanisms of virus inhibition
Source: PLoS Pathog. 2026 Jun 5;22(6):e1014268. doi: 10.1371/journal.ppat.1014268 (PMC13262937; doi:10.1371/journal.ppat.1014268)
Supplement: S4 Table — Table summarizes the data collection, image processing, and model refinement statistics for the structures of the complex of BG505 SOSIP.664, a chaperone PGT121 Fab, and the Fabs of four mAbs isolated from RUp16. (DOCX) [file ppat.1014268.s015.docx]

**S4 Table. Cryo-EM data collection and refinement statistics.**

|  | **1G3 Fab-PGT 121 Fab-BG505 SOSIP.664 complex** | **1A8 Fab-PGT 121 Fab-BG505 SOSIP.664 complex** | **4 Fab-PGT 121 Fab-BG505 SOSIP.664 complex** | **76 Fab-PGT 121 Fab-BG505 SOSIP.664 complex** |
| --- | --- | --- | --- | --- |
| EMDB | 72216 | 72713 | 72206 | 72094 |
| PDB | 9Q3X | 9Y9V | 9Q3S | 9Q09 |
| **Data Collection and Processing** | | | | |
| Microscope | FEI Glacios | Talos Arctica | JEOL CRYO ARM 200 | JEOL CRYO ARM 200 |
| Voltage (kV) | 200 | 200 | 200 | 200 |
| Detector | Gatan K3 | Gatan K3 | Gatan K3 | Gatan K3 |
| Pixel Size (Å/px) | 0.8893 | 0.7185 | 0.9213 | 0.9213 |
| Total electron dose (e^-^/Å^2^) | 60 | 46 | 60 | 60 |
| Defocus Range (µm) | 0.5-2.7 | 0.5-2.7 | 0.5-2.0 | 0.5-2.0 |
| Micrograph collected | 2,756 | 977 | 7,620 | 6,849 |
| Number of picked particles | 1,124,000 | 1,119,152 | 2,392,293 | 5,717,622 |
| Number of final particles | 425,978 | 349,369 | 436,694 | 332,541 |
| Symmetry | C3 | C3 | C3 | C1 |
| Resolution (Å) (FSC 0.143) | 3.64 | 2.98 | 3.01 | 3.23 |
| **Refinement (Phenix) & validation** | | | | |
| Model composition |  |  |  |  |
| Protein | 24,117 | 25,361 | 25,975 | 24,729 |
| glycan | 1,773 | 580 | 664 | 2,207 |
| Average B-factors (Å^2^) |  |  |  |  |
| Protein | 79 | 77 | 67 | 95 |
| glycan | 76 | 78 | 79 | 116 |
| CC_mask | 0.75 | 0.80 | 0.80 | 0.86 |
| EMRinger Score | 1.08 | 2.35 | 2.89 | 2.4 |
| RMSD Bond lengths (Å) | 0.002 | 0.003 | 0.003 | 0.006 |
| RMSD Bond angles (˚) | 0.58 | 0.65 | 0.66 | 0.84 |
| Molprobity score | 2.17 | 2.21 | 2.44 | 2.52 |
| Clash score | 6.3 | 6.65 | 7.8 | 7.70 |
| Ramachandran Plot |  |  |  |  |
| Favored (%) | 94.6 | 94.2 | 93.3 | 92.1 |
| Allowed (%) | 5.4 | 5.7 | 6.4 | 7.7 |
| Disallowed (%) | 0.0 | 0.1 | 0.3 | 0.3 |

Table summarizes the data collection, image processing, and model refinement statistics for the structures of the complex of BG505 SOSIP.664, a chaperone PGT121 Fab, and the Fabs of four mAbs isolated from RUp16.
