# Supplementary material for: HIV-1 BG505 SOSIP immunization induced B cell expansion targeting the 465-glycan hole, with neutralizing antibodies exhibiting distinct binding modes and mechanisms of virus inhibition
Source: PLoS Pathog. 2026 Jun 5;22(6):e1014268. doi: 10.1371/journal.ppat.1014268 (PMC13262937; doi:10.1371/journal.ppat.1014268)
Supplement: Attachment — Submitted filename: Myers PLoS Path_IB_TC.docx [file ppat.1014268.s016.docx]

Summary

This manuscript by Myers, Chandravanshi and colleagues includes thorough characterization of BG505 SOSIP.664-induced neutralizing clonotypes isolated from macaques with high neutralization titers after multiple autologous immunizations. Cryo-EM structures of some monoclonal antibodies (mAbs) show that these antibodies all target the non-conserved BG505-specific 456 glycan hole region, with one antibody also approaching the CD4bs region. Neutralization capacity of neutralizing antibodies is restricted to the BG505 strain.

As the authors extensively unfold the binding mechanisms of BG505-neutralizing mAbs, they argue that it is unlikely that immunizations with untargeted HIV immunogens will induce neutralization breadth, consistent with existing literature and the current dogma. It is valuable to characterize clonotypes / mAbs that will likely never develop to become broadly neutralizing, as this can be informative to sequence analysis as well as in the context of epitope competition / antibody feedback. Therefore, although this is not always made explicit in the way the authors present this work, this paper includes meaningful data for the HIV vaccine field.

Major comments:

1. In the way this work is presented, it is currently opaque what this study contributes to what is already known. Previous publications by the same group have reported on immunization studies with fully glycosylated Envelope trimers and strain-specific immunodominant glycan holes – two of the structures presented in the current manuscript stem from that study.
2. Throughout the manuscript, pivotal data that is being referred to is in the supplement and I recommend carefully revising what is considered essential for the story and what is actually supplementary. Examples are neutralization data (figure S5) and gene distributions (figure S2). Moreover, statements such as “the N462Q BG505 was much more resistance than wildtype to neutralization …” should be supported with IC50s (and neutralization curves). Other examples include CDRH3 distributions could be interesting since this is mentioned later (line 275). How do 9 AA long CDRH3s compare to the entire BG505 SOSIP.664 induced repertoire? Are such short 9 AA CDRH3s also present in the human repertoire? Could the authors consider adding such information to the main body of the manuscript?
3. In Figure 1A, the authors report on clone sizes, normalized by the number of productive BCR sequences and the number of clones per timepoint. By doing so, the figure becomes very abstract and difficult to interpret. Could the authors comment on what is defined as medium and what is large? How many cells? Also, how many clones are included at each timepoint? This figure requires more information and more resolution. The authors could consider adding it to the supplement or defining more bins for small-medium-large clonotypes.
4. Line 148: The authors conclude that B cell diversity following repeated immunization decreases based on intra-clonal expansion and increased mutations. But whereas clonal expansion could indeed decrease B cell diversity, SHM actually increases B cell diversity. Can the authors comment? Or is there a better way show that B cell diversity decreases?
5. Figure 2A: With competition assays, does it matter which antibody is presented first given that higher affinity antibodies could better compete with lower affinity antibodies than *vice versa*? Also, could the authors consider to expand the selection of CD4bs bnAbs, as CD4bs-targeting bnAbs can have very different footprints.
6. The authors indicate that mAb 1A8 engages with loop D residue T278 (Fig 4), but the structure in Fig 3 does not suggest that any light chain loop is in close proximity with loop D. How is BSA determined? This should be added to the methods. Also, could (1) the authors show side views of the structures so that it is becomes clear how these mAbs target Env, (2) could the authors provide a comparison with CD4bs bnAbs to show how different these mAbs are from CD4bs-targeting bnAbs and (3) upon introduction of the structures, could the authors mention whether it regards cryo-EM structures or crystal structures? Moreover, I could not access PDBs from the portal but there are options to give reviewers access to PDBs before publication to verify the authors’ analyses.
7. The language throughout the paper can be somewhat sensationalist in tone. For example, these are not amongst the first cryo-EM structures from rhesus macaque mAbs. Please also revise to remove “interestingly”/”notably” etc., or “potent” when neutralization is not particularly potent.
8. The authors focus on the 465 glycan hole. However, a Scripps group published in 2021 on cryo-EMPEM analyses of BG505 SOSIP-immunized NHPs and define another important glycan hole: the 241/289 glycan holes. It is mentioned in the main text that these classes of 465-targetin mAbs reconstitute the serum neutralization of these animals well. Considering that the sorting probe was the less-than-ideal BG505 gp120 instead of the full trimer, how can the authors be sure that ONLY this class of nAbs is responsible for serum neutralization? The 241/289 GH is also very immunogenic and leads to strain-specific neutralization. Please also discuss this glycan hole as the work now is being presented as if 465 is the only glycan hole on BG505 SOSIP.

Minor comments:

1. mAb 76 has the most mutated heavy chain (17% mutated?, Table 1) and happens to be the only imaged mAb that contacts more conserved CD4b loop. How do the mutated residues contribute towards the BSA within that region? Are there any signs for selection of mutations that enhance CD4b loop engagement?
2. Line 211: The authors indicate that “a glycan at N462 partially obstructed epitope access, as is the case for VRC01”. The difference here seems to be very small, especially as there can be quite some variation between neutralization experiments. Could the authors show data on both experiments to show that this effect is consistently observed?
3. Why did the authors choose to use N462Q to measure sensitivity to the 465 glycan hole and not the previously used construct T465N?
4. Figure 3C and 5D: text is too small.
5. Line 173: The fact that clonotype 559 mAbs bound poorly could also be because of trimer “breathing”, not necessarily dissociation, or simply interference with other trimer regions not present on gp120. I recommend refraining from too much speculation in the results section.
6. Line 188-189: It could also be that these mAbs simply do not neutralize?
7. Why was clonotype 599 excluded from competition analysis because figure S4A shows little difference in binding between clonotype 599 and 385.
8. What is the target antigen of the negative control, EM4C04?
9. Line 318-319: The authors indicate that there are smaller changes in binding kinetics of mab 4 to wildtype and glycan-trimmed SOSIPs, compared to 1G3 and 1A8. But the KD fold change of mab 4 is 7, which is only 2-fold less than 1A8 and 1G3 have. How significant is this difference?
10. The title of the paper is a whopping 27 words. Could the authors consider shortening the title?
