# Supplementary material for: HIV-1 BG505 SOSIP immunization induced B cell expansion targeting the 465-glycan hole, with neutralizing antibodies exhibiting distinct binding modes and mechanisms of virus inhibition
Source: PLoS Pathog. 2026 Jun 5;22(6):e1014268. doi: 10.1371/journal.ppat.1014268 (PMC13262937; doi:10.1371/journal.ppat.1014268)
Supplement: Attachment — Submitted filename: Response to reviewers_final.docx [file ppat.1014268.s017.docx]

**Part I -Summary**

Reviewer #1: This manuscript by Myers, Chandravanshi and colleagues includes thorough characterization of BG505 SOSIP.664-induced neutralizing clonotypes isolated from macaques with high neutralization titers after multiple autologous immunizations. Cryo-EM structures of some monoclonal antibodies (mAbs) show that these antibodies all target the non-conserved BG505-specific 456 glycan hole region, with one antibody also approaching the CD4bs region. Neutralization capacity of neutralizing antibodies is restricted to the BG505 strain. As the authors extensively unfold the binding mechanisms of BG505-neutralizing mAbs, they argue that it is unlikely that immunizations with untargeted HIV immunogens will induce neutralization breadth, consistent with existing literature and the current dogma. It is valuable to characterize clonotypes / mAbs that will likely never develop to become broadly neutralizing, as this can be informative to sequence analysis as well as in the context of epitope competition / antibody feedback. Therefore, although this is not always made explicit in the way the authors present this work, this paper includes meaningful data for the HIV vaccine field.

We thank reviewer #1 for their strong endorsement of our work.

Reviewer #2: This manuscript presents an in-depth follow-up to the authors’ previously published NHP work (Refs. 5 and 10). The detailed tracing of Ag+ B-cell expansion of the major clonal types, together with several cryo-EM structures of mAb/SOSIP complexes, adds substantial mechanistic detail and helps explain the functional properties of these antibodies. Overall, I think this is a strong and useful study, and I support publication after revision.

We thank reviewer #2 for their recognition of the importance of our work.

Part II – Major Issues: Key Experiments Required for Acceptance

Reviewer #1:

1. In the way this work is presented, it is currently opaque what this study contributes to what is already known. Previous publications by the same group have reported on immunization studies with fully glycosylated Envelope trimers and strain-specific immunodominant glycan holes – two of the structures presented in the current manuscript stem from that study.

The reviewer is not correct in stating that two of the structures we present ‘stem from a previous study’. First, we have not published or alluded to any structures of these antibodies in prior studies. Second, the initial characterization of only one antibody in this paper, 1G3, was described in the previous publication by Charles et al. 2021. Antibody 1A8 was not included in our prior study and was isolated later from a different sample and time point (40 weeks later). We described these facts in the original text.

Furthermore, the extensive longitudinal B cell receptor sequencing and analysis, isolation and characterization of new neutralizing and non-neutralizing monoclonal antibodies of multiple clonotypes and time points, and the four cryo-EM structures of neutralizing antibodies bound to BG505 SOSIP are new and have not been published in our prior papers. The serum neutralization titers presented for RUp16 in supplementary material overlap with those presented in our previous publications, but we considered it necessary to highlight those in the current study given their significance.

Finally, this work is a new collaboration between Dr. Derdeyn and Dr. Pazgier, who have not published together before. In summary, this manuscript is novel and presents new insight into BG505 SOSIP immunogenicity, glycan hole targeting, mechanisms of antibody neutralization, and antigen specific clonal dynamics underlying high serum neutralization titers.

2. Throughout the manuscript, pivotal data that is being referred to is in the supplement and I recommend carefully revising what is considered essential for the story and what is actually supplementary. Examples are neutralization data (figure S5) and gene distributions (figure S2). Moreover, statements such as “the N462Q BG505 was much more resistance than wildtype to neutralization …” should be supported with IC50s (and neutralization curves). Other examples include CDRH3 distributions could be interesting since this is mentioned later (line 275). How do 9 AA long CDRH3s compare to the entire BG505 SOSIP.664 induced repertoire? Are such short 9 AA CDRH3s also present in the human repertoire?

We have moved figures S2 and S5 to the main text as requested. They are now figures 1 and 3, respectively. The IC50 values for all Env-antibody combinations are included in Table 1, which was already present in the original main text of the manuscript. We have included BG505 Env T465N pseudovirus in these datasets, as requested below by the reviewer.

We have added figure 1E to show that there were ~40 antigen specific clonotypes with a 9 amino-acid CDRH3. Overall, the number of clonotypes carrying CDRH3s of varying lengths follows the same distribution of CDRH3 lengths shown in the original manuscript, now shown in figure 1C. Short CDRH3s (such as 9 aa) were not common or enriched. CDRH3s of 9 aa length are found in humans in both naïve and antigen experienced B cells, as presented in this paper (Sankar, Hon Hoi, Hotzel, Nature Communications, 2020, PMID: 32358517). They are not exclusive to rhesus macaques. We have added this information and reference to the paper.

3. Could the authors consider adding such information to the main body of the manuscript?

We have added the information to the revised manuscript as described above.

4. In Figure 1A, the authors report on clone sizes, normalized by the number of productive BCR sequences and the number of clones per timepoint. By doing so, the figure becomes very abstract and difficult to interpret. Could the authors comment on what is defined as medium and what is large? How many cells? Also, how many clones are included at each timepoint? This figure requires more information and more resolution. The authors could consider adding it to the supplement or defining more bins for small-medium-large clonotypes.

We have replaced figure 1A with a new figure (now figure 2A) that displays the number of clonotypes that fall into three size categories (small, medium, and large) on different graphs and better defines the categories by percentage of cells included in a given clonotype at each time point. We hope this is clearer for the reviewer and readers. The main points are that singlets/small clonotypes are always dominant but medium and large clonotypes arise with subsequent immunizations and then the large clonotypes wane. The number of cells corresponding to each time point is now shown in figure 2B, and this information was included in the original manuscript in figure 1B. We have modified that section of the text as appropriate.

5. Line 148: The authors conclude that B cell diversity following repeated immunization decreases based on intra-clonal expansion and increased mutations. But whereas clonal expansion could indeed decrease B cell diversity, SHM actually increases B cell diversity. Can the authors comment? Or is there a better way show that B cell diversity decreases?

To make these points more clearly, we have modified the text as follows: “Thus, B cell clonotype diversity at the antigen specific population level declined progressively as intra-clonal expansion and mutation away from germline increased intra-lineage diversity with repeated immunizations.”

6. Figure 2A: With competition assays, does it matter which antibody is presented first given that higher affinity antibodies could better compete with lower affinity antibodies than *vice versa*? Also, could the authors consider to expand the selection of CD4bs bnAbs, as CD4bs-targeting bnAbs can have very different footprints.

We performed the competition assay in both directions, and the residual binding was averaged. We have added this information to the BLI methods section and to the legend for this figure.

As the reviewer suggested, we also performed competition experiments with the CD4bs bnAb CH103, selected because it has a different footprint from the VRC01-like bnAbs (i.e. CDRH3 dominant, does not engage N276). This data is now included in Fig. 4 and discussed in the related text. CH103 and VRC01 produced similar competition profiles.

7. The authors indicate that mAb 1A8 engages with loop D residue T278 (Fig 4), but the structure in Fig 3 does not suggest that any light chain loop is in close proximity with loop D. How is BSA determined? This should be added to the methods. Also, could (1) the authors show side views of the structures so that it is becomes clear how these mAbs target Env, (2) could the authors provide a comparison with CD4bs bnAbs to show how different these mAbs are from CD4bs-targeting bnAbs and (3) upon introduction of the structures, could the authors mention whether it regards cryo-EM structures or crystal structures? Moreover, I could not access PDBs from the portal but there are options to give reviewers access to PDBs before publication to verify the authors’ analyses.

We thank the reviewer for his/her insight on this manuscript. Engagement of loop D residue T278 by mAb 1A8 was assessed through buried surface area (BSA) calculations using the PDBePISA web server. This algorithm employs a standard 1.4 Å probe radius to approximate a water molecule rolling over the protein surface. Details of the BSA calculations have now been added to the methods section (see the new methods section on buried surface area calculations). In response to the reviewer’s suggestion, we have included additional side views of the structures (new panel Figure 5C), which more clearly illustrate the spatial relationship between the antibody CDRs from the light chain and gp120 from Env protein.

We have also expanded our analysis to include BSA comparisons with representative CD4 binding-site (CD4bs) broadly neutralizing antibodies, including VRC01, 04_A06, VRC13, 3BNC117 and CH103. These comparisons are now included in the revised manuscript (Revised Fig. 6, S10 and S11).

We have revised the text to specify that the structures were determined using cryo-electron microscopy (cryo-EM) in multiple places. Finally, the coordinates have been deposited in the RCSB Protein Data Bank and are currently on hold until publication. We have notified the editor that the PDB files can be shared or made available to reviewers upon request to facilitate evaluation of our analyses.

8. The language throughout the paper can be somewhat sensationalist in tone. For example, these are not amongst the first cryo-EM structures from rhesus macaque mAbs. Please also revise to remove “interestingly”/”notably” etc., or “potent” when neutralization is not particularly potent.

The reviewer has misquoted our text. We said that “The results are among the first cryo-EM structures of nAbs elicited by BG505 SOSIP.664 in RM, and they reveal distinct binding modes, navigation of the surrounding glycans to reach the underlying protein surface, variable proximity to the CD4bs, and strain-specific Env contacts, sometimes alongside more conserved elements, that limited neutralization to BG505.” We did not claim in any way that they our antibodies are “amongst the first cryo-EM structures from rhesus macaque mAbs”.

Regarding our use of interestingly/notably, we queried ChatGPT to ask how frequently the BG505 SOSIP peer-reviewed literature uses interpretive adverbs. It found that they are used heavily, and ‘interestingly’ and ‘notably’ were among the most common interpretive adverbs and phrases that appeared in peer-reviewed BG505 SOSIP papers on PubMed (#2, #3, respectively). This information therefore argues that these words are not ‘sensationalist’ but are used commonly to highlight or emphasize an interpretation or detail in peer reviewed papers on similar topics.

We also queried ChatGPT to investigate use of the term neutralization potency in peer reviewed BG505 SOSIP papers on PubMed. This is the information it provided. There is **no universal standard**, but many papers studying broadly neutralizing antibodies (bnAbs) use ranges such as: extremely potent <0.01ug/ml; very potent 0.01-0.1 ug/ml, potent 0.1-1 ug/ml, moderately potent 1-10 ug/ml, and >10 ug/ml weak or non-potent. As a specific example, a paper by Caniels et al. describes ‘potent neutralizing activity’ for antibodies with IC50 titers ranging from 0.003-15 ug/ml against an N276 glycan mutant (Science 2025, PMID 40373114). Our use of the term potent to describe the neutralization we report for our antibodies is in line with the conventions of the field and in fact some of our antibodies would be described as ‘very potent’ according to these conventions.

We also note in three of our previous peer-reviewed published PLoS Pathogens papers (Charles, 2021; Welbourn 2022; Mopuri 2023), we used the terms interestingly, notable, and notably on multiple occasions. We also described antibodies with IC50s similar to those reported here as being potent.

9. The authors focus on the 465 glycan hole. However, a Scripps group published in 2021 on cryo-EMPEM analyses of BG505 SOSIP-immunized NHPs and define another important glycan hole: the 241/289 glycan holes. It is mentioned in the main text that these classes of 465-targetin mAbs reconstitute the serum neutralization of these animals well. Considering that the sorting probe was the less-than-ideal BG505 gp120 instead of the full trimer, how can the authors be sure that ONLY this class of nAbs is responsible for serum neutralization? The 241/289 GH is also very immunogenic and leads to strain-specific neutralization. Please also discuss this glycan hole as the work now is being presented as if 465 is the only glycan hole on BG505 SOSIP.

In the Charles et al. paper, which we referenced, we mapped the serum neutralization targets of RUp16 and also performed cryo-EMPEM. For serum neutralization, the results indicated that the major contributor to the potent serum neutralization in this animal was the 465 glycan hole, which we state in the original manuscript and provide our reference. If you go back to that paper, you can see that we did not detect any contribution by the 241/289 glycan hole in that experiment for RUp16. This is despite there being 241/289 glycan hole antibodies circulating in RUp16 serum as detected by cryo-EMPEM, also shown in the referenced paper. It is important to note that cryo-EMPEM detects both neutralizing and non-neutralizing specificities, as we described in that paper. Other BG505 SOSIP animals in the cohort did have serum neutralizing antibodies that targeted the 241/289 glycan hole, as shown in the referenced paper, but those are not included here. For RUp16, the 465 glycan hole was the dominant neutralizing target.

We have modified the text to include Klasse 2018 reference for the 241/289 glycan hole in rhesus macaques, and that 241/289 was not targeted by serum neutralizing antibodies in RUp16 although antibodies against this region were detected by cryo-EMPEM, referencing our previous paper.

Reviewer #2:

1. The authors did not provide enough explanation for why these antibodies are sequence-specific. The structural data are useful, but some additional sequence analysis would help clarify this point.

We thank the reviewer for this suggestion. In the revised manuscript, we have included a sequence conservation analysis (Fig. S11). In this figure, the molecular surface of gp120 is displayed and colored according to sequence conservation. In addition, the epitopes of the mAbs identified in this study, along with representative CD4-binding site Ab footprints, are mapped onto the surface to enable direct comparison. The highlighted regions indicate the specific residues engaged by each antibody, allowing visualization of the relationship between epitope location and sequence conservation.

2. The lack of structural studies on the non-neutralizing mAbs is a weakness. Some of these antibodies appear to bind the SOSIP trimer well, so structural information on at least representative non-neutralizing mAbs would help explain why they fail to neutralize. If this is beyond the scope of the current study, the authors should at least discuss this limitation more directly.

We thank the reviewer for the suggestion. We agree this would be an interesting and valuable direction. However, this is beyond the scope for this paper. We will consider exploring this in future work.

**Part III – Minor Issues: Editorial and Data Presentation Modifications**

Reviewer #1:

1. mAb 76 has the most mutated heavy chain (17% mutated?, Table 1) and happens to be the only imaged mAb that contacts more conserved CD4b loop. How do the mutated residues contribute towards the BSA within that region? Are there any signs for selection of mutations that enhance CD4b loop engagement?

We examined longitudinal sequence variation within the 1303 clonotype (119 paired heavy and light chain sequences), considering the residues in the Env BG505 CD4 binding loop region (I358, R360, F361, A362, N363, S364, S365, G366, G367) that were involved in contact with mAb 76 (E33, Y58, G99, S100, W100A, I100B in heavy; S54, D54A in light). Only position 54 in the light chain showed any variation, with S54G appearing in 11 sequences across time points. The remaining residues that directly contact the CD4 binding loop in mAb 76 are completely conserved. Residue S54 in CDRL2 contributes only approximately 6% (36 Å^2^) of the light chain BSA contribution to the interface. To put things in context, CDRL1, CDRL2, and CDRL3 contribute approximately 214, 114, and 294 Å^2^ to the light chain BSA, respectively. This indicates strong conservation of the CDR residues responsible for contacting the CD4 binding loop, rather than ongoing affinity maturation to enhance this engagement. As is evident from the divergence from germline, there are mutations/toggling in in many other positions. This analysis suggests that recognition of the CD4 binding loop was established early in the lineage and maintained throughout maturation, while somatic mutation primarily affected other regions of the antibody that may contribute to overall stability, orientation, or contacts outside the CD4 binding loop. We included this information in the discussion.

2. Line 211: The authors indicate that “a glycan at N462 partially obstructed epitope access, as is the case for VRC01”. The difference here seems to be very small, especially as there can be quite some variation between neutralization experiments. Could the authors show data on both experiments to show that this effect is consistently observed?

Our neutralization curves show the average of **a minimum of** two independent experiments, with bars indicating error/variation on all graphs. All independent experiments are averaged for the reported IC50s. VRC01 was analyzed independently 5 times against the WT and the 462Q mutant in the TZM-bl assay. The variation of those 5 experiments is captured in the error bars (standard error of the mean) on the graph, which are very small. It would be unconventional to show individual graphs for 5 independent experiments. Below are the infectivity curves for the 5 independent VRC01 experiments. There are consistent differences in infectivity at the two highest concentrations, which is exactly what is shown on the graph, and produced modestly different IC50 values (0.10 ug/ml for WT, 0.06 ug/ml for N462Q). We have added the data for VRC01 against the T465N mutant pseudovirus (0.11 ug/ml), which was run twice, and there is a consistent shift for N462Q but not T465N shown in Fig. 3A (and Table 1). Nevertheless, we have removed the statement ‘as is the case for VRC01’ as requested by the reviewer.

3. Why did the authors choose to use N462Q to measure sensitivity to the 465 glycan hole and not the previously used construct T465N?

As we stated in our previous paper that is referenced (Charles et al., 2021), the previous RUp16 mAbs tested for neutralization of T465N including 1G3 ‘exhibited different capacities to neutralize the T465N mutant’ while they had additional indicators of glycan hole recognition (N398A, T357A, TI356KT) that were included in that paper. Our observations suggested that ‘the absence or presence of glycans adjacent to residue 465 is not the only determinant of neutralization and is consistent with the heterogeneity within this epitope and among the antibodies directed towards it’. We therefore originally chose to use N462Q as an indicator of proximity to the CD4 binding site, citing references that used it as such, as the competition assays and the cryo-EM structures placed the epitopes in or near the 465 glycan hole.

Nevertheless, we have tested the antibody panel against T465N in the TZM-bl assay. mAbs from the 684 clonotype (including 1G3 and 1A8) showed variable sensitivity to T465N, consistent with the results presented in the Charles et al paper. The increase in IC50 ranged from 2.1-fold (1A8) to 163-fold (32). 1G3 was similar to our previous study (12- vs 25-fold increase in IC50). The other neutralizing mAbs had modest increases in IC50 against T465N from 2- to 6-fold. Non-neutralizing mAbs were not affected by the T465N mutation.

The T465N data is included in the new Fig. 3A, Table 1, and discussed in the associated text.

4. Figure 3C and 5D: text is too small.

Figure 3C (now Figure 5D) and Figure 5D (now Figure 7D) have been recreated with improved font size (please see the revised figures).

5. Line 173: The fact that clonotype 559 mAbs bound poorly could also be because of trimer “breathing”, not necessarily dissociation, or simply interference with other trimer regions not present on gp120. I recommend refraining from too much speculation in the results section.

We have modified the text to “mAbs from five of the six clonotypes also bound comparably to BG505 SOSIP.664; however, clonotype 599 mAbs bound poorly to the SOSIP.”

6. Line 188-189: It could also be that these mAbs simply do not neutralize?

The clonotype 695 mAbs do neutralize BG505 Env pseudovirus and T465N. The reviewer’s comment is unclear.

7. Why was clonotype 599 excluded from competition analysis because figure S4A shows little difference in binding between clonotype 599 and 385.

In the binding kinetics experiments shown in Fig. S4A (now Fig. S3A), gp120 and the SOSIP were in solution and the mAbs were bound to the sensor. Conversely, in the competition assay, the gp120 protein was immobilized to a Ni-NTA sensor and the mAbs were in solution. As we explained in the original manuscript in the competition results section, the mAbs from clonotype 599 bound poorly to BG505 gp120 in the sensor immobilized configuration and were therefore excluded from the competition analysis. We have also added this statement at the end of the figure legend (new figure 4).

8. What is the target antigen of the negative control, EM4C04?

We stated in the original manuscript in the BLI methods section that EM4C04 is “a human antibody specific for 2009 pH1N1 virus hemagglutinin” and provided the reference. We have added ‘anti-flu HA’ to the legends for figures 3 and 4.

9. Line 318-319: The authors indicate that there are smaller changes in binding kinetics of mab 4 to wildtype and glycan-trimmed SOSIPs, compared to 1G3 and 1A8. But the KD fold change of mab 4 is 7, which is only 2-fold less than 1A8 and 1G3 have. How significant is this difference?

We thank the reviewer for this important point. The experiment was performed in triplicate (n = 3), and each independent run showed a consistent fold difference, supporting the reproducibility of the results. The purpose of this experiment was to compare the change in binding affinity of each monoclonal antibody between glycosylated and deglycosylated BG505 SOSIPs. The fold change in binding affinity between glycosylated and deglycosylated SOSIPs was consistent for each mAb and significant. However, mAb 4 exhibited a lower fold change compared to mAbs 1A8 and 1G3, indicating relatively smaller sensitivity to glycan removal.

10. The title of the paper is a whopping 27 words. Could the authors consider shortening the title?

We shortened the title to 24 words.

Reviewer #2:

1. The Results section was hard to read in places and could be organized more clearly.

We have made many revisions to the manuscript that we believe will make the results clearer.

- We have moved two supplementary figures into the main text showing information about the antigen specific B cell population (Fig. 1) and the neutralization curves, which now contain an additional BG505 mutant, T465N (Fig. 3).
- We replaced Fig. 1A with a new figure (now Fig. 2A) that we hope is clearer and have modified the text.
- We added another CD4bs bnAb to the competition assay (CH103) (Fig. 4).
- We included an additional BG505 mutant to the neutralization assays (T465N) (Fig. 3A and Table 1).
- We improved the visual quality of the structural figures (Fig. 5 and 7).
- We added information on other CD4bs bnAb epitopes for comparison (Fig. 6 and S10).
- We added a figure showing the conservation of the epitopes for our antibodies across HIV-1 (Fig. S11).
- We added color indicators of the IC50s in Table 1.
- We have added clarifying information and details in multiple places.

We believe that these changes will make the results section clearer and better organized for the reader.

2. The name of the animal studied, RUp16, should be mentioned when first introduced in the Introduction.

We have added RUp16 to the introduction when first mentioned.

3. The PDB ID 9QX3 should be corrected to 9Q3X.

Thank you for catching this. We apologize for this typo and the ID has been corrected.

4. In Table S1, the group size could be included in the Vaccine group column for distinction from the weeks.

Thank you for this suggestion to improve the clarity of the table. We have made this modification to Table S1.
