# Supplementary material for: HIV-1 BG505 SOSIP immunization induced B cell expansion targeting the 465-glycan hole, with neutralizing antibodies exhibiting distinct binding modes and mechanisms of virus inhibition
Source: PLoS Pathog. 2026 Jun 5;22(6):e1014268. doi: 10.1371/journal.ppat.1014268 (PMC13262937; doi:10.1371/journal.ppat.1014268)
Supplement: Attachment — Submitted filename: Response to Reviewers May 2026.docx [file ppat.1014268.s018.docx]

**Please see our responses to reviewer #1 below.**

**Part I - Summary**

Reviewer #1: I thank the authors for their careful evaluation of the comments. Most comments are addressed (more than) appropriately. Several (small) improvements are to be made:

Reviewer #2: (No Response)

**Part II – Major Issues: Key Experiments Required for Acceptance**

Reviewer #1:

1. There is no doubt this manuscript is important to the field. However, some directions/clarifications throughout the paper were missing, especially some comments on where this work fits into the current dogma of the HIV vaccine field. It is currently believed that fully glycosylated immunogens are not likely to induce bnAbs - although the authors acknowledge this in the manuscript, it could be emphasized more how this data contributes to the HIV field knowing that these antibodies will likely never be boosted to become broad. Could they be auxiliary nAbs to the bnAbs induced by some other trimer? The data presented is relevant, as this information can help to identify which B cell lineages NOT to chase, but the authors could clarify this as this would highlight the importance of the authors’ work better. The way the work is currently presented could be confusing for readers within the HIV vaccine field, and complicates interpretation for those outside the HIV field.

We have modified and added the following text to the end of the discussion:

Strategies are needed to improve the efficiency of repeated immunizations to drive the intra-clonal expansion, recall, and mutation, that appears to be associated with high titer, durable neutralization responses while incorporating more precise epitope targeting and elements of viral diversity. The strain specific epitopes we identified are proximal to the CD4bs, a bnAb target, and even include a conserved CD4bs element in the case of mAb 76. It is possible that the neutralizing clonotypes we characterized here could have acquired breadth if they were exposed to other trimers besides BG505 or immunogens with missing or rearranged glycans, a strategy employed by others to drive heterologous neutralization breadth (37, 41-46).

2. We appreciate the authors for adjusting figure 1 and 2 - the figures are clearer to interpret and seem more complete. I was aware that the IC50 values were present in Table 1, but to increase interpretability while reading, I would recommend to add them in the main text as well.

The IC50 values/ranges of the main neutralizing antibodies/clonotypes discussed in the results section were already added to the main text when this was requested at the last review.

“Clonotype 684 was first detected at week 26, and the mAb from that time point (mAb 18) neutralized BG505 Env pseudovirus with an IC50 titer of 0.24 ug/mL despite having 98% nucleotide identity to germline (Table 1;Fig. 3A). Neutralization potency increased 5-fold between weeks 26 and 93 to 0.05 ug/mL, along with substantial divergence from both VH and VL germline alleles. For clonotype 695, mAb 70 isolated from week 26 was weakly neutralizing (Table 1; Fig.3A). The clonotype 695 mAbs from weeks 48 and 81 neutralized more potently with higher divergence from germline, reaching IC50 titers of 0.39 and 0.42 ug/mL, respectively. Additionally, the neutralization curve of the clonotype 695 antibodies 4 and 12 against wild type (WT) BG505 and T465N exhibited a plateau that can be caused by factors such as virion heterogeneity, low stoichiometry, and high off antibody rates (2, 19, 20) (Fig. 3A). For the third neutralizing clonotype, 1303, only one mAb was tested, from week 81. This mAb 76 had an IC50 titer of 0.21 ug/mL”

As for CDRH3 lengths of 9aa: this reviewer is aware of them being present in human naïve and memory repertoires. I would argue that the distribution in Fig. 1E is not a normal distribution, and there are statistical tests to test for that. The occurrence of 9aa CDRH3s is also considerably higher than is shown in the paper that the authors refer to (Sankar, Hon Hoi, Hotzel, Nature Communications, 2020, PMID: 32358517). Therefore, please add some statement in the Results or Discussion section pertaining to the likelihood of this length occurring in human repertoires.

We have modified and added the following text:

We have updated the figure legends and text for Figs. 1C and 1E to distinguish between the frequency across all cells vs. the number of clonotypes.

In the results:

The length distribution of the third complementarity determining regions for V_H_ and V_L_, CDRH3 and CDRL3 across all cells, are shown in **Fig. 1C** and **1D**, respectively. The number of clonotypes that had CDRH3 or CDRL3 regions of a given length are shown in **Fig. 1E** and **1F**, respectively. Overall, the CDRH3 lengths from antigen specific B cells in this one immunized animal followed a similar distribution to antigen naïve and experienced human CDRH3 lengths (19). The median CDRH3 length was 15 amino acids, ranging from 7 to 32 amino acids, while the CDRL3 lengths were more restricted (7 to 15 amino acids, median length of 11 amino acids).

In the discussion:

The best neutralizing mAbs were from clonotype 684, with the shortest CDRH3 of 9 amino acids. However, short CDRH3 lengths, such as 9 amino acids, were not common or enriched in the Ag+ B cell population, and were present at a frequency of 2% (Fig. 1E). B cell receptors with such short CDRH3 lengths are also found in humans in both naïve and antigen experienced populations at similar frequencies to what we observed here (19).

3/4/5/6/7/9. Good improvements to the manuscript and thanks for including additional experiments on CH103 and showing additional BSA comparisons for the structural data.

8. Regardless of whether the quote was correct or not, the sentence “The results are among the first cryo-EM structures of nAbs elicited by BG505 SOSIP.664 in RM” is simply not correct. BG505 SOSIP.664 has been used >10 years now in RMs and many nAbs and admittedly, non-nAbs, have been characterized. As I intended with my comment, I would urge caution expressing terms such as “the first”, “novel” etcetera. Reputable journals such as Science and its sister journals explicitly include “Please avoid statements of future work, claims of priority, and repetition of conclusions at the end.” in their submission templates, and it is good practice to avoid those expletives. Similarly, Springer Nature wrote an editorial >20 years ago ([https://www.nature.com/articles/ni1105-1061](https://urldefense.com/v3/__https:/www.nature.com/articles/ni1105-1061__;!!K-Hz7m0Vt54!nSgFjMj5e8DoJguwbJGhl_k3gmvU-vXo6zqs2qTrYGnC6W-TsyCNNa2GSUzMsYoHT8WBYU0DBMciiQ$)), touching on both the use of “novel” (“…as in claims of primacy such as “we are the first to demonstrate” or “this highly novel result.” Again, if in fact the data are truly novel, readers will not require prompting to think so.”) and “interestingly” (“For example, 'remarkably', 'interestingly', 'surprisingly' and similar adverbs are very often used with the aim of providing emphasis, but their actual function is to persuade—something that has no place in scientific manuscripts—rather than to convey information. If the data are indeed remarkable, readers will not need prompting to believe so.”).

While I applaud creative use of generative AI, I am not sure it holds enough value to justify the authors’ explanation for the use of “interestingly”. The fact that others (and indeed, as the authors say, the authors themselves) use these expletives does not mean it is correct. I realize this may be a case of ‘agree to disagree’, but given the authors’ enthusiasm in using ChatGPT, maybe Google’s Gemini can convince them to reconsider words such as “interestingly”: “In academic writing, ‘interestingly’ is often considered a filler word or an unnecessary adverb that should be avoided in favor of objective analysis. It is generally used to highlight surprising results, but it can make writing appear subjective or desperate to force attention, whereas strong data should speak for itself.”

Regarding the use of “potent”, this reviewer agrees that it is not a universal standard. However, the scientific standards mentioned above should apply here (i.e., better to be careful with expletives), and instances of “unusually potent/high” throughout the manuscript could be changed.

The phrase “The results are among the first…” has been removed from the text.

The words potent, potently, interestingly, notably, unusually, remarkably, and exceptionally, etc. have been removed from the text.

Reviewer #2: (No Response)

**Part III – Minor Issues: Editorial and Data Presentation Modifications**

Reviewer #1:

1. It is interesting to read that this lineage had an inherent advantage for CD4bs recognition, rather than acquiring it through SHM over time, and a great addition to the manuscript.

2-9. No further comments, we appreciate the authors for elaborating on their experiments.

10. This reviewer would argue 24 words is still too long for a title.

Reviewer #2: (No Response)
