# Supplementary material for: HIV-1 BG505 SOSIP immunization induced B cell expansion targeting the 465-glycan hole, with neutralizing antibodies exhibiting distinct binding modes and mechanisms of virus inhibition
Source: PLoS Pathog. 2026 Jun 5;22(6):e1014268. doi: 10.1371/journal.ppat.1014268 (PMC13262937; doi:10.1371/journal.ppat.1014268)
Supplement: Peer review history

# Peer review: Decision Letter 0

**DOI:** 10.1371/journal.ppat.1014268.r001

**Authors:** Richard A. Koup, Penny L. Moore

16 Mar 2026

-->PPATHOGENS-D-26-00266

HIV-1 BG505 SOSIP immunization induced expansion of multiple B cell clonotypes against the 465-glycan hole, with neutralizing antibodies exhibiting distinct binding modes and mechanisms of virus inhibition

PLOS Pathogens

Dear Dr. Derdeyn,

Thank you for submitting your manuscript to PLOS Pathogens. After careful consideration, we feel that it has merit but does not fully meet PLOS Pathogens's publication criteria as it currently stands. Therefore, we invite you to submit a revised version of the manuscript that addresses the points raised during the review process.

Please submit your revised manuscript by May 15 2026 11:59PM. If you will need more time than this to complete your revisions, please reply to this message or contact the journal office at plospathogens@plos.org. When you're ready to submit your revision, log on to https://www.editorialmanager.com/ppathogens/ and select the 'Submissions Needing Revision' folder to locate your manuscript file.

Please include the following items when submitting your revised manuscript:

* A letter that responds to each point raised by the editor and reviewer(s). You should upload this letter as a separate file labeled 'Response to Reviewers'. This file does not need to include responses to any formatting updates and technical items listed in the 'Journal Requirements' section below.

* A marked-up copy of your manuscript that highlights changes made to the original version. You should upload this as a separate file labeled 'Revised Manuscript with Track Changes'.

* An unmarked version of your revised paper without tracked changes. You should upload this as a separate file labeled 'Manuscript'.

If you would like to make changes to your financial disclosure, competing interests statement, or data availability statement, please make these updates within the submission form at the time of resubmission. Guidelines for resubmitting your figure files are available below the reviewer comments at the end of this letter.

We look forward to receiving your revised manuscript.

Kind regards,

Penny L. Moore

Academic Editor

PLOS Pathogens

Richard Koup

Section Editor

PLOS Pathogens

Sumita Bhaduri-McIntosh

Editor-in-Chief

PLOS Pathogens

orcid.org/0000-0003-2946-9497

Michael Malim

Editor-in-Chief

PLOS Pathogens

orcid.org/0000-0002-7699-2064

**Journal Requirements:**

1) Please ensure that the CRediT author contributions listed for every co-author are completed accurately and in full.

At this stage, the following Authors/Authors require contributions: August Myers, Monika Chandravanshi, Leanne S Whitmore, Brendan F Kohrn, Amina Negash, Dung N Nguyen, Pooja Ralli-Jain, Kendra Cruickshank, Amit A Upadhyay, Tysheena Charles, Christopher T Edwards, Eric Hunter, Rama R Amara, Marek K Korseniowski, Ling Niu, Edwin Pozharski, William D Tolbert, Steven E Bosinger, Scott R Kennedy, Marzena Pazgier, and Cynthia A Derdeyn. Please ensure that the full contributions of each author are acknowledged in the "Add/Edit/Remove Authors" section of our submission form.

The list of CRediT author contributions may be found here: https://journals.plos.org/plospathogens/s/authorship#loc-author-contributions

2) We ask that a manuscript source file is provided at Revision. Please upload your manuscript file as a .doc, .docx, .rtf or .tex. If you are providing a .tex file, please upload it under the item type u2018LaTeX Source Fileu2019 and leave your .pdf version as the item type u2018Manuscriptu2019.

3) Please upload all main figures as separate Figure files in .tif or .eps format. For more information about how to convert and format your figure files please see our guidelines:

https://journals.plos.org/plospathogens/s/figures

4) Thank you for providing your data availability statement. We noted that the datasets with the following accession numbers "EMD-72216  EMD-72713 , EMD-72206 and EMD- 72094" are held till publication. Please note that, though access restrictions are acceptable now, your entire data will need to be made freely accessible if your manuscript is accepted for publication. This policy applies to all data except where public deposition would breach compliance with the protocol approved by your research ethics board.

5) Please amend your detailed Financial Disclosure statement. This is published with the article. It must therefore be completed in full sentences and contain the exact wording you wish to be published.

1) State what role the funders took in the study. If the funders had no role in your study, please state: "The funders had no role in study design, data collection and analysis, decision to publish, or preparation of the manuscript."

6) Please ensure that the funders and grant numbers match between the Financial Disclosure field and the Funding Information tab in your submission form. Note that the funders must be provided in the same order in both places as well. Currently, "Emory Consortium for Innovative AIDS Research in Nonhuman Primates" is missing from the Funding Information tab.

7) Thank you for stating that "The 10X VDJ sequence data has been deposited into the Sequence Read Archive (SRA) under accession number PRJNA1357351 and at https://dataview.ncbi.nlm.nih.gov/object/PRJNA1357351?reviewer=vuvo52u9v7oadmtmbfk27ipo5d. All codes used for analysis in this paper can be found at https://github.com/Derdeyn-Lab/Myers_intra-clonal-expansion_of_antibodies_that_neutralize_HIV." Please ensure that the provided links are working.

Note: If the reviewer comments include a recommendation to cite specific previously published works, please review and evaluate these publications to determine whether they are relevant and should be cited. There is no requirement to cite these works unless the editor has indicated otherwise.

**Reviewers' Comments:**

**Comments to the Authors:**

**Please note that one review is uploaded as an attachment.**

Reviewer's Responses to Questions

**Part I - Summary**

Please use this section to discuss strengths/weaknesses of study, novelty/significance, general execution and scholarship.

Reviewer #1: This manuscript by Myers, Chandravanshi and colleagues includes thorough characterization of BG505 SOSIP.664-induced neutralizing clonotypes isolated from macaques with high neutralization titers after multiple autologous immunizations. Cryo-EM structures of some monoclonal antibodies (mAbs) show that these antibodies all target the non-conserved BG505-specific 456 glycan hole region, with one antibody also approaching the CD4bs region. Neutralization capacity of neutralizing antibodies is restricted to the BG505 strain.

As the authors extensively unfold the binding mechanisms of BG505-neutralizing mAbs, they argue that it is unlikely that immunizations with untargeted HIV immunogens will induce neutralization breadth, consistent with existing literature and the current dogma. It is valuable to characterize clonotypes / mAbs that will likely never develop to become broadly neutralizing, as this can be informative to sequence analysis as well as in the context of epitope competition / antibody feedback. Therefore, although this is not always made explicit in the way the authors present this work, this paper includes meaningful data for the HIV vaccine field.

Reviewer #2: This manuscript presents an in-depth follow-up to the authors’ previously published NHP work (Refs. 5 and 10). The detailed tracing of Ag+ B-cell expansion of the major clonal types, together with several cryo-EM structures of mAb/SOSIP complexes, adds substantial mechanistic detail and helps explain the functional properties of these antibodies. Overall, I think this is a strong and useful study, and I support publication after revision.

**********

**Part II – Major Issues: Key Experiments Required for Acceptance**

Please use this section to detail the key new experiments or modifications of existing experiments that should be absolutely required to validate study conclusions.

Generally, there should be no more than 3 such required experiments or major modifications for a "Major Revision" recommendation. If more than 3 experiments are necessary to validate the study conclusions, then you are encouraged to recommend "Reject".

Reviewer #1: Major comments:

1. In the way this work is presented, it is currently opaque what this study contributes to what is already known. Previous publications by the same group have reported on immunization studies with fully glycosylated Envelope trimers and strain-specific immunodominant glycan holes – two of the structures presented in the current manuscript stem from that study.

2. Throughout the manuscript, pivotal data that is being referred to is in the supplement and I recommend carefully revising what is considered essential for the story and what is actually supplementary. Examples are neutralization data (figure S5) and gene distributions (figure S2). Moreover, statements such as “the N462Q BG505 was much more resistance than wildtype to neutralization …” should be supported with IC50s (and neutralization curves). Other examples include CDRH3 distributions could be interesting since this is mentioned later (line 275). How do 9 AA long CDRH3s compare to the entire BG505 SOSIP.664 induced repertoire? Are such short 9 AA CDRH3s also present in the human repertoire? Could the authors consider adding such information to the main body of the manuscript?

3. In Figure 1A, the authors report on clone sizes, normalized by the number of productive BCR sequences and the number of clones per timepoint. By doing so, the figure becomes very abstract and difficult to interpret. Could the authors comment on what is defined as medium and what is large? How many cells? Also, how many clones are included at each timepoint? This figure requires more information and more resolution. The authors could consider adding it to the supplement or defining more bins for small-medium-large clonotypes.

4. Line 148: The authors conclude that B cell diversity following repeated immunization decreases based on intra-clonal expansion and increased mutations. But whereas clonal expansion could indeed decrease B cell diversity, SHM actually increases B cell diversity. Can the authors comment? Or is there a better way show that B cell diversity decreases?

5. Figure 2A: With competition assays, does it matter which antibody is presented first given that higher affinity antibodies could better compete with lower affinity antibodies than vice versa? Also, could the authors consider to expand the selection of CD4bs bnAbs, as CD4bs-targeting bnAbs can have very different footprints.

6. The authors indicate that mAb 1A8 engages with loop D residue T278 (Fig 4), but the structure in Fig 3 does not suggest that any light chain loop is in close proximity with loop D. How is BSA determined? This should be added to the methods. Also, could (1) the authors show side views of the structures so that it is becomes clear how these mAbs target Env, (2) could the authors provide a comparison with CD4bs bnAbs to show how different these mAbs are from CD4bs-targeting bnAbs and (3) upon introduction of the structures, could the authors mention whether it regards cryo-EM structures or crystal structures? Moreover, I could not access PDBs from the portal but there are options to give reviewers access to PDBs before publication to verify the authors’ analyses.

7. The language throughout the paper can be somewhat sensationalist in tone. For example, these are not amongst the first cryo-EM structures from rhesus macaque mAbs. Please also revise to remove “interestingly”/”notably” etc., or “potent” when neutralization is not particularly potent.

8. The authors focus on the 465 glycan hole. However, a Scripps group published in 2021 on cryo-EMPEM analyses of BG505 SOSIP-immunized NHPs and define another important glycan hole: the 241/289 glycan holes. It is mentioned in the main text that these classes of 465-targetin mAbs reconstitute the serum neutralization of these animals well. Considering that the sorting probe was the less-than-ideal BG505 gp120 instead of the full trimer, how can the authors be sure that ONLY this class of nAbs is responsible for serum neutralization? The 241/289 GH is also very immunogenic and leads to strain-specific neutralization. Please also discuss this glycan hole as the work now is being presented as if 465 is the only glycan hole on BG505 SOSIP.

Reviewer #2: 1. The authors did not provide enough explanation for why these antibodies are sequence-specific. The structural data are useful, but some additional sequence analysis would help clarify this point.

2. The lack of structural studies on the non-neutralizing mAbs is a weakness. Some of these antibodies appear to bind the SOSIP trimer well, so structural information on at least representative non-neutralizing mAbs would help explain why they fail to neutralize. If this is beyond the scope of the current study, the authors should at least discuss this limitation more directly.

**********

**Part III – Minor Issues: Editorial and Data Presentation Modifications**

Please use this section for editorial suggestions as well as relatively minor modifications of existing data that would enhance clarity.

Reviewer #1: Minor comments:

1. mAb 76 has the most mutated heavy chain (17% mutated?, Table 1) and happens to be the only imaged mAb that contacts more conserved CD4b loop. How do the mutated residues contribute towards the BSA within that region? Are there any signs for selection of mutations that enhance CD4b loop engagement?

2. Line 211: The authors indicate that “a glycan at N462 partially obstructed epitope access, as is the case for VRC01”. The difference here seems to be very small, especially as there can be quite some variation between neutralization experiments. Could the authors show data on both experiments to show that this effect is consistently observed?

3. Why did the authors choose to use N462Q to measure sensitivity to the 465 glycan hole and not the previously used construct T465N?

4. Figure 3C and 5D: text is too small.

5. Line 173: The fact that clonotype 559 mAbs bound poorly could also be because of trimer “breathing”, not necessarily dissociation, or simply interference with other trimer regions not present on gp120. I recommend refraining from too much speculation in the results section.

6. Line 188-189: It could also be that these mAbs simply do not neutralize?

7. Why was clonotype 599 excluded from competition analysis because figure S4A shows little difference in binding between clonotype 599 and 385.

8. What is the target antigen of the negative control, EM4C04?

9. Line 318-319: The authors indicate that there are smaller changes in binding kinetics of mab 4 to wildtype and glycan-trimmed SOSIPs, compared to 1G3 and 1A8. But the KD fold change of mab 4 is 7, which is only 2-fold less than 1A8 and 1G3 have. How significant is this difference?

10. The title of the paper is a whopping 27 words. Could the authors consider shortening the title?

Reviewer #2: 1. The Results section was hard to read in places and could be organized more clearly.

2. The name of the animal studied, RUp16, should be mentioned when first introduced in the Introduction.

3. The PDB ID 9QX3 should be corrected to 9Q3X.

4. In Table S1, the group size could be included in the Vaccine group column for distinction from the weeks.

**********

PLOS authors have the option to publish the peer review history of their article (what does this mean?). If published, this will include your full peer review and any attached files.

If you choose “no”, your identity will remain anonymous but your review may still be made public.

**Do you want your identity to be public for this peer review?**   For information about this choice, including consent withdrawal, please see our Privacy Policy.

Reviewer #1: No

Reviewer #2: No

[NOTE: If reviewer comments were submitted as an attachment file, they will be attached to this email and accessible via the submission site. Please log into your account, locate the manuscript record, and check for the action link "View Attachments". If this link does not appear, there are no attachment files.]

**Figure resubmission:**

-->While revising your submission, we strongly recommend that you use PLOS’s NAAS tool (https://ngplosjournals.pagemajik.ai/artanalysis) to test your figure files. NAAS can convert your figure files to the TIFF file type and meet basic requirements (such as print size, resolution), or provide you with a report on issues that do not meet our requirements and that NAAS cannot fix.

After uploading your figures to PLOS’s NAAS tool - https://ngplosjournals.pagemajik.ai/artanalysis, NAAS will process the files provided and display the results in the "Uploaded Files" section of the page as the processing is complete. If the uploaded figures meet our requirements (or NAAS is able to fix the files to meet our requirements), the figure will be marked as "fixed" above. If NAAS is unable to fix the files, a red "failed" label will appear above. When NAAS has confirmed that the figure files meet our requirements, please download the file via the download option, and include these NAAS processed figure files when submitting your revised manuscript.

**Reproducibility:**

To enhance the reproducibility of your results, we recommend that authors of applicable studies deposit laboratory protocols in protocols.io, where a protocol can be assigned its own identifier (DOI) such that it can be cited independently in the future. Additionally, PLOS ONE offers an option to publish peer-reviewed clinical study protocols. Read more information on sharing protocols at https://plos.org/protocols?utm_medium=editorial-email&utm_source=authorletters&utm_campaign=protocols

Attachment Submitted filename: Myers PLoS Path_IB_TC.docx

# Author comment: Author response to Decision Letter 1

**DOI:** 10.1371/journal.ppat.1014268.r002

7 Apr 2026

Attachment Submitted filename: Response to reviewers_final.docx

# Peer review: Decision Letter 1

**DOI:** 10.1371/journal.ppat.1014268.r003

**Authors:** Richard A. Koup, Penny L. Moore

11 May 2026

PPATHOGENS-D-26-00266R1

HIV-1 BG505 SOSIP immunization induced B cell expansion targeting the 465-glycan hole, with neutralizing antibodies exhibiting distinct binding modes and mechanisms of virus inhibition

PLOS Pathogens

Dear Dr. Derdeyn,

Thank you for submitting your manuscript to PLOS Pathogens. After careful consideration, we feel that it has merit but does not fully meet PLOS Pathogens's publication criteria as it currently stands. Therefore, we invite you to submit a revised version of the manuscript that addresses the points raised during the review process. I am comfortable leaving the title as it is, despite the suggestion by reviewer 1. Please address all other comments, including those around language, so that we can move forward with a decision.

Please submit your revised manuscript by Jul 10 2026 11:59PM. If you will need more time than this to complete your revisions, please reply to this message or contact the journal office at plospathogens@plos.org. When you're ready to submit your revision, log on to https://www.editorialmanager.com/ppathogens/ and select the 'Submissions Needing Revision' folder to locate your manuscript file.

Please include the following items when submitting your revised manuscript:

* A letter that responds to each point raised by the editor and reviewer(s). You should upload this letter as a separate file labeled 'Response to Reviewers'. This file does not need to include responses to any formatting updates and technical items listed in the 'Journal Requirements' section below.

* A marked-up copy of your manuscript that highlights changes made to the original version. You should upload this as a separate file labeled 'Revised Manuscript with Track Changes'.

* An unmarked version of your revised paper without tracked changes. You should upload this as a separate file labeled 'Manuscript'.

If you would like to make changes to your financial disclosure, competing interests statement, or data availability statement, please make these updates within the submission form at the time of resubmission. Guidelines for resubmitting your figure files are available below the reviewer comments at the end of this letter.

As the corresponding author, your ORCID iD is verified in the submission system and will appear in the published article. PLOS supports the use of ORCID, and we encourage all coauthors to register for an ORCID iD and use it as well. Please encourage your coauthors to verify their ORCID iD within the submission system before final acceptance, as unverified ORCID iDs will not appear in the published article. Only the individual author can complete the verification step; PLOS staff cannot verify ORCID iDs on behalf of authors.

We look forward to receiving your revised manuscript.

Kind regards,

Penny L. Moore

Academic Editor

PLOS Pathogens

Richard Koup

Section Editor

PLOS Pathogens

Sumita Bhaduri-McIntosh

Editor-in-Chief

PLOS Pathogens

orcid.org/0000-0003-2946-9497

Michael Malim

Editor-in-Chief

PLOS Pathogens

orcid.org/0000-0002-7699-2064

**Journal Requirements:**

If the reviewer comments include a recommendation to cite specific previously published works, please review and evaluate these publications to determine whether they are relevant and should be cited. There is no requirement to cite these works unless the editor has indicated otherwise.

1) Thank you for including an Ethics Statement for your study. Please include:

i) The approval number(s), or a statement that approval was granted by the named board(s).

2) In the online submission form, you indicated that your data will be submitted to a repository upon acceptance. We strongly recommend all authors deposit their data before acceptance, as the process can be lengthy and hold up publication timelines. Please note that, though access restrictions are acceptable now, your entire minimal dataset will need to be made freely accessible if your manuscript is accepted for publication. This policy applies to all data except where public deposition would breach compliance with the protocol approved by your research ethics board. If you are unable to adhere to our open data policy, please kindly revise your statement to explain your reasoning and we will seek the editor's input on an exemption.

**Reviewers' Comments:**

Reviewer's Responses to Questions

**Part I - Summary**

Please use this section to discuss strengths/weaknesses of study, novelty/significance, general execution and scholarship.

Reviewer #1: I thank the authors for their careful evaluation of the comments. Most comments are addressed (more than) appropriately. Several (small) improvements are to be made:

Reviewer #2: (No Response)

**********

**Part II – Major Issues: Key Experiments Required for Acceptance**

Please use this section to detail the key new experiments or modifications of existing experiments that should be absolutely required to validate study conclusions.

Generally, there should be no more than 3 such required experiments or major modifications for a "Major Revision" recommendation. If more than 3 experiments are necessary to validate the study conclusions, then you are encouraged to recommend "Reject".

Reviewer #1: 1. There is no doubt this manuscript is important to the field. However, some directions/clarifications throughout the paper were missing, especially some comments on where this work fits into the current dogma of the HIV vaccine field. It is currently believed that fully glycosylated immunogens are not likely to induce bnAbs - although the authors acknowledge this in the manuscript, it could be emphasized more how this data contributes to the HIV field knowing that these antibodies will likely never be boosted to become broad. Could they be auxiliary nAbs to the bnAbs induced by some other trimer? The data presented is relevant, as this information can help to identify which B cell lineages NOT to chase, but the authors could clarify this as this would highlight the importance of the authors’ work better. The way the work is currently presented could be confusing for readers within the HIV vaccine field, and complicates interpretation for those outside the HIV field.

2. We appreciate the authors for adjusting figure 1 and 2 - the figures are clearer to interpret and seem more complete. I was aware that the IC50 values were present in Table 1, but to increase interpretability while reading, I would recommend to add them in the main text as well.

As for CDRH3 lengths of 9aa: this reviewer is aware of them being present in human naïve and memory repertoires. I would argue that the distribution in Fig. 1E is not a normal distribution, and there are statistical tests to test for that. The occurrence of 9aa CDRH3s is also considerably higher than is shown in the paper that the authors refer to (Sankar, Hon Hoi, Hotzel, Nature Communications, 2020, PMID: 32358517). Therefore, please add some statement in the Results or Discussion section pertaining to the likelihood of this length occurring in human repertoires.

3/4/5/6/7/9. Good improvements to the manuscript and thanks for including additional experiments on CH103 and showing additional BSA comparisons for the structural data.

8. Regardless of whether the quote was correct or not, the sentence “The results are among the first cryo-EM structures of nAbs elicited by BG505 SOSIP.664 in RM” is simply not correct. BG505 SOSIP.664 has been used >10 years now in RMs and many nAbs and admittedly, non-nAbs, have been characterized. As I intended with my comment, I would urge caution expressing terms such as “the first”, “novel” etcetera. Reputable journals such as Science and its sister journals explicitly include “Please avoid statements of future work, claims of priority, and repetition of conclusions at the end.” in their submission templates, and it is good practice to avoid those expletives. Similarly, Springer Nature wrote an editorial >20 years ago (https://www.nature.com/articles/ni1105-1061), touching on both the use of “novel” (“…as in claims of primacy such as “we are the first to demonstrate” or “this highly novel result.” Again, if in fact the data are truly novel, readers will not require prompting to think so.”) and “interestingly” (“For example, 'remarkably', 'interestingly', 'surprisingly' and similar adverbs are very often used with the aim of providing emphasis, but their actual function is to persuade—something that has no place in scientific manuscripts—rather than to convey information. If the data are indeed remarkable, readers will not need prompting to believe so.”).

While I applaud creative use of generative AI, I am not sure it holds enough value to justify the authors’ explanation for the use of “interestingly”. The fact that others (and indeed, as the authors say, the authors themselves) use these expletives does not mean it is correct. I realize this may be a case of ‘agree to disagree’, but given the authors’ enthusiasm in using ChatGPT, maybe Google’s Gemini can convince them to reconsider words such as “interestingly”: “In academic writing, ‘interestingly’ is often considered a filler word or an unnecessary adverb that should be avoided in favor of objective analysis. It is generally used to highlight surprising results, but it can make writing appear subjective or desperate to force attention, whereas strong data should speak for itself.”

Regarding the use of “potent”, this reviewer agrees that it is not a universal standard. However, the scientific standards mentioned above should apply here (i.e., better to be careful with expletives), and instances of “unusually potent/high” throughout the manuscript could be changed.

Reviewer #2: (No Response)

**********

**Part III – Minor Issues: Editorial and Data Presentation Modifications**

Please use this section for editorial suggestions as well as relatively minor modifications of existing data that would enhance clarity.

Reviewer #1: 1. It is interesting to read that this lineage had an inherent advantage for CD4bs recognition, rather than acquiring it through SHM over time, and a great addition to the manuscript.

2-9. No further comments, we appreciate the authors for elaborating on their experiments.

10. This reviewer would argue 24 words is still too long for a title.

Reviewer #2: (No Response)

**********

PLOS authors have the option to publish the peer review history of their article (what does this mean?). If published, this will include your full peer review and any attached files.

If you choose “no”, your identity will remain anonymous but your review may still be made public.

**Do you want your identity to be public for this peer review?**   For information about this choice, including consent withdrawal, please see our Privacy Policy.

Reviewer #1: No

Reviewer #2: No

[NOTE: If reviewer comments were submitted as an attachment file, they will be attached to this email and accessible via the submission site. Please log into your account, locate the manuscript record, and check for the action link "View Attachments". If this link does not appear, there are no attachment files.]

**Figure resubmission:**

While revising your submission, we strongly recommend that you use PLOS’s NAAS tool (https://ngplosjournals.pagemajik.ai/artanalysis) to test your figure files. NAAS can convert your figure files to the TIFF file type and meet basic requirements (such as print size, resolution), or provide you with a report on issues that do not meet our requirements and that NAAS cannot fix.

After uploading your figures to PLOS’s NAAS tool - https://ngplosjournals.pagemajik.ai/artanalysis, NAAS will process the files provided and display the results in the "Uploaded Files" section of the page as the processing is complete. If the uploaded figures meet our requirements (or NAAS is able to fix the files to meet our requirements), the figure will be marked as "fixed" above. If NAAS is unable to fix the files, a red "failed" label will appear above. When NAAS has confirmed that the figure files meet our requirements, please download the file via the download option, and include these NAAS processed figure files when submitting your revised manuscript.

**Reproducibility:**

To enhance the reproducibility of your results, we recommend that authors of applicable studies deposit laboratory protocols in protocols.io, where a protocol can be assigned its own identifier (DOI) such that it can be cited independently in the future. Additionally, PLOS ONE offers an option to publish peer-reviewed clinical study protocols. Read more information on sharing protocols at https://plos.org/protocols?utm_medium=editorial-email&utm_source=authorletters&utm_campaign=protocols

# Author comment: Author response to Decision Letter 2

**DOI:** 10.1371/journal.ppat.1014268.r004

13 May 2026

Attachment Submitted filename: Response to Reviewers May 2026.docx

# Editor report: Decision Letter 2

**DOI:** 10.1371/journal.ppat.1014268.r005

**Authors:** Richard A. Koup, Penny L. Moore

15 May 2026

Dear Dr. Derdeyn,

We are pleased to inform you that your manuscript 'HIV-1 BG505 SOSIP immunization induced B cell expansion targeting the 465-glycan hole, with neutralizing antibodies exhibiting distinct binding modes and mechanisms of virus inhibition' has been provisionally accepted for publication in PLOS Pathogens.

Before your manuscript can be formally accepted you will need to complete some formatting changes, which you will receive in a follow up email. A member of our team will be in touch with a set of requests.

Please note that your manuscript will not be scheduled for publication until you have made the required changes, so a swift response is appreciated.

IMPORTANT: The editorial review process is now complete. PLOS will only permit corrections to spelling, formatting or significant scientific errors from this point onwards. Requests for major changes, or any which affect the scientific understanding of your work, will cause delays to the publication date of your manuscript.

Should you, your institution's press office or the journal office choose to press release your paper, you will automatically be opted out of early publication. We ask that you notify us now if you or your institution is planning to press release the article. All press must be co-ordinated with PLOS.

Thank you again for supporting Open Access publishing; we are looking forward to publishing your work in PLOS Pathogens.

Best regards,

Penny L. Moore

Academic Editor

PLOS Pathogens

Susan Ross

Section Editor

PLOS Pathogens

Sumita Bhaduri-McIntosh

Editor-in-Chief

PLOS Pathogens

orcid.org/0000-0003-2946-9497

Michael Malim

Editor-in-Chief

PLOS Pathogens

orcid.org/0000-0002-7699-2064

***********************************************************

Reviewer Comments (if any, and for reference):

# Editor report: Acceptance letter

**DOI:** 10.1371/journal.ppat.1014268.r006

**Authors:** Richard A. Koup, Penny L. Moore

Dear Dr. Derdeyn,

We are delighted to inform you that your manuscript, "HIV-1 BG505 SOSIP immunization induced B cell expansion targeting the 465-glycan hole, with neutralizing antibodies exhibiting distinct binding modes and mechanisms of virus inhibition," has been formally accepted for publication in PLOS Pathogens.

We have now passed your article onto the PLOS Production Department who will complete the rest of the pre-publication process. All authors will receive a confirmation email upon publication.

The corresponding author will soon be receiving a typeset proof for review, to ensure errors have not been introduced during production. Please review the PDF proof of your manuscript carefully, as this is the last chance to correct any scientific or type-setting errors. Please note that major changes, or those which affect the scientific understanding of the work, will likely cause delays to the publication date of your manuscript. Note: Proofs for Front Matter articles (Pearls, Reviews, Opinions, etc...) are generated on a different schedule and may not be made available as quickly.

Soon after your final files are uploaded, the early version of your manuscript, if you opted to have an early version of your article, will be published online. The date of the early version will be your article's publication date. The final article will be published to the same URL, and all versions of the paper will be accessible to readers.

For Research Articles, you will receive an invoice from PLOS for your publication fee after your manuscript has reached the completed accept phase. If you receive an email requesting payment before acceptance or for any other service, this may be a phishing scheme. Learn how to identify phishing emails and protect your accounts at https://explore.plos.org/phishing.

Thank you again for supporting open-access publishing; we are looking forward to publishing your work in PLOS Pathogens.

Best regards,

Sumita Bhaduri-McIntosh

Editor-in-Chief

PLOS Pathogens

orcid.org/0000-0003-2946-9497

Michael Malim

Editor-in-Chief

PLOS Pathogens

orcid.org/0000-0002-7699-2064
